# Reversibly Sticking Metals and Graphite to Hydrogels and Tissues

**DOI:** 10.1021/acscentsci.3c01593

**Published:** 2024-03-13

**Authors:** Wenhao Xu, Faraz A. Burni, Srinivasa R. Raghavan

**Affiliations:** † Department of Chemistry & Biochemistry, University of Maryland, College Park, Maryland 20742, United States; ‡ Department of Chemical & Biomolecular Engineering, University of Maryland, College Park, Maryland 20742, United States

## Abstract

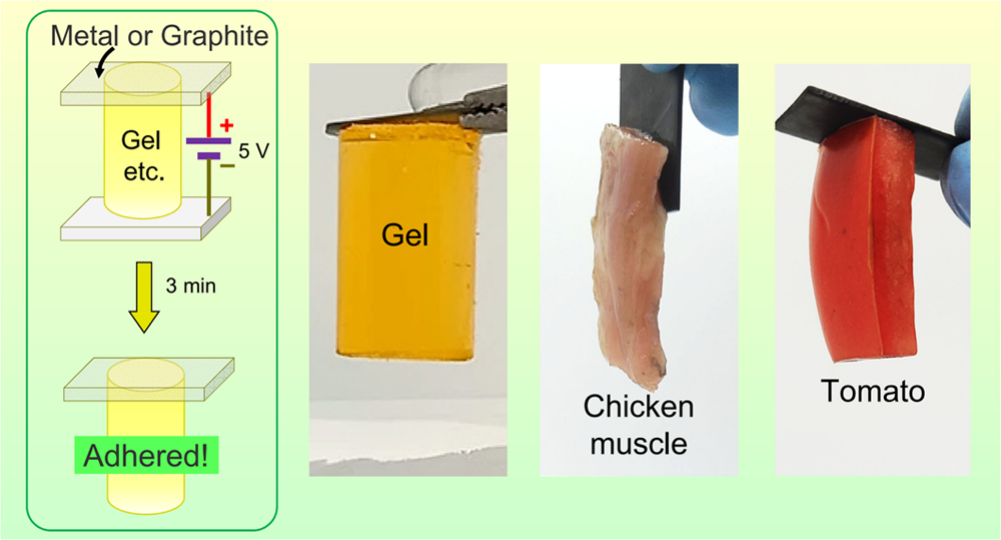

We have discovered that hard, electrical conductors (e.g., metals or graphite) can be adhered to soft, aqueous materials (e.g., hydrogels, fruit, or animal tissue) without the use of an adhesive. The adhesion is induced by a low DC electric field. As an example, when 5 V DC is applied to graphite slabs spanning a tall cylindrical gel of acrylamide (AAm), a strong adhesion develops between the anode (+) and the gel in about 3 min. This adhesion endures after the field is removed, and we term it as *hard–soft electroadhesion* or **EA**^[HS]^. Depending on the material, adhesion occurs at the anode (+), cathode (−), or both electrodes. In many cases, **EA**^[HS]^ can be reversed by reapplying the field with reversed polarity. Adhesion via **EA**^[HS]^ to AAm gels follows the electrochemical series: e.g., it occurs with copper, lead, and tin but not nickel, iron, or zinc. We show that **EA**^[HS]^ arises via electrochemical reactions that generate chemical bonds between the electrode and the polymers in the gel. **EA**^[HS]^ can create new hybrid materials, thus enabling applications in robotics, energy storage, and biomedical implants. Interestingly, **EA**^[HS]^ can even be achieved underwater, where typical adhesives cannot be used.

## Abstract 2

A DC electric field can be used to stick metals or graphite to soft materials including gels, animal tissues, fruits, and vegetables. Such electroadhesion is reversible and even works underwater.

## Introduction

This work is concerned with both soft and hard solids. Common examples of soft solids are *hydrogels*, which are three-dimensional networks of polymer chains swollen with water.^1−3^ The networks can be cross-linked by chemical (covalent) bonds or physical (noncovalent) bonds such as hydrogen bonds. Examples of the latter include gelatin gels, which are a popular dessert (Jell-O) in many parts of the world.^4,5^ Other examples of soft, aqueous materials include plant products (fruits and vegetables), aquatic animals, and the tissues in our body.^6,7^ Indeed, typical biological cells are soft and gel-like, with a water content around 70%.^7,8^ Yet, the architecture of vertebrate animals, including humans, shows the need to combine soft tissues with hard structural elements, i.e., bones, vertebrae, and the skeleton.^7,9^ The hard elements are needed to support the weight of the animal, provide structural integrity, and transmit force. The need to combine soft and hard elements also comes into the fore when designing soft robots or actuators.^10,11^ Researchers have realized that, for a robot to exert forces, soft, force-generating elements (akin to muscle) must be interfaced with stiff, load-bearing elements (akin to bone) via elements of intermediate stiffness (akin to cartilage).^10^

Motivated by some of the points mentioned above, several researchers have attempted to adhere soft hydrogels to hard solids (e.g., metals, plastic, wood, glass).^12−20^ However, to achieve adhesion, typically the chemistry of either the hydrogel or the hard surface must be modified. One common modification has been inspired by the chemistry of mussels and is via catechol groups.^21−23^ Mussels stick to rocky surfaces by secreting filaments rich in catechols, which form strong coordination bonds with the surfaces. Accordingly, catechols can be introduced into gel backbones to make gels adhere to hard surfaces.^22,23^ Other chemistries have also been exploited to induce strong gel–solid adhesion. For instance, Sekine et al.^15^ functionalized acrylamide (AAm) gels with azide groups and contacted the gels with glass surfaces decorated with alkyne groups. A cycloaddition reaction between the azides and alkynes ensued, leading to a strong adhesion. In most of the above cases where a gel is bonded to a hard solid, the adhesion is permanent; i.e., the two cannot be easily detached at a later time if needed. Thus, to summarize the literature, gel–solid adhesion has been achieved mostly for chemically tailored gels or surfaces, and once the gel is adhered to the solid, their adhesion is generally permanent and irreversible.

Recently, there has also been interest in triggering adhesion (i.e., achieving “adhesion on command”) via an external stimulus such as electric fields.^24−26^ For example, a DC field can induce adhesion between a cationic and an anionic gel.^24^ When the gels are subjected to 10 V DC for ∼10 s, they become stuck, and the adhesion endures after the field is removed. This gel-gel *electroadhesion* (EA) is believed to be due to the electrophoretic migration of polymer chains across the gel-gel interface. In a similar vein, we have shown that cationic gels can be electroadhered to animal tissues, which are known to be anionic.^25^ This gel-tissue EA again has all the above hallmarks (it is a permanent adhesion induced by 10 V DC within seconds). Interestingly, both gel-gel and gel-tissue EA can be reversed at a later point simply by placing the adhered pair under 10 V DC and reversing the polarity. Thus, EA occurs between soft, aqueous materials of opposite charge and is strong, durable, and reversible.

Electric fields have also been used to stick hard and soft materials, but such adhesion typically decays and disappears quickly when the field is switched off.^27−31^ For example, a metal can be stuck to a soft dielectric material (such as an elastomer or a nonaqueous gel) when a DC field >120 V is applied across the pair.^31^ This phenomenon is also called electroadhesion, but it is conceptually very different and has an electrostatic origin (i.e., the Johnson-Rahbek effect^29^). When the field is turned off, the materials quickly lose this electrostatic adhesion; thus, this phenomenon allows metallic grippers in a robot to pick up objects and then release them.^27,31^ Note that this electrostatic effect cannot lead to permanent adhesion in the absence of the field. To our knowledge, the only example of enduring hard–soft adhesion induced by an electric field was the recent study of Qiu et al.^32^ The authors contacted a hydrogel with glass as well as two iron electrodes and applied a DC field of ∼6 V for several hours. The glass (but not the electrodes) adhered to the gel, and this was attributed to the formation of iron(III) hydroxide nanoparticles at the gel-glass interface. This finding seems to be restricted to a particular choice of electrodes (and to thin gels) and, therefore, is not generalizable.

**Figure 1:**
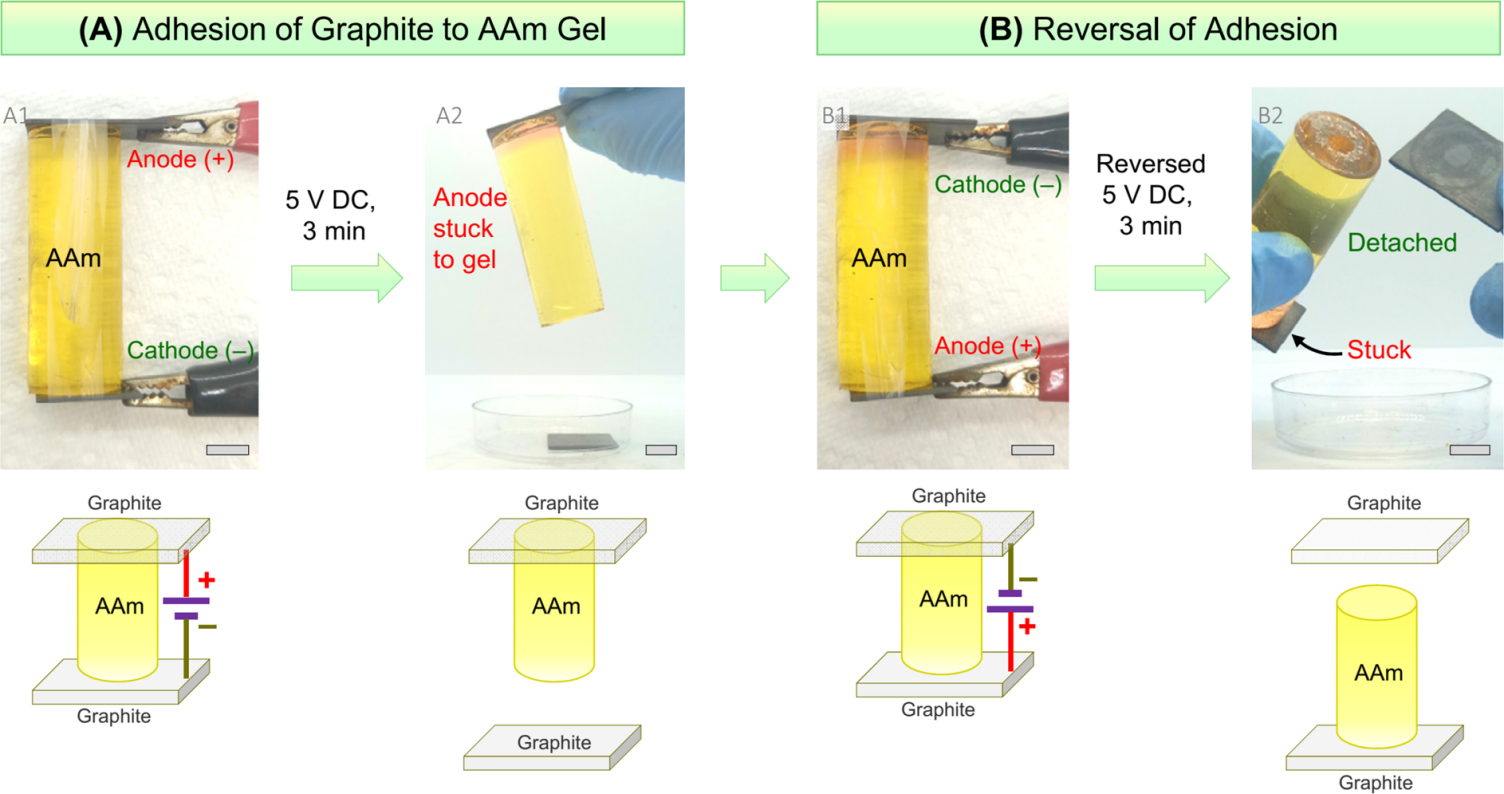
Reversible electroadhesion of graphite to an acrylamide (AAm) hydrogel. Photos and schematics are shown for each case. First, graphite slabs are placed on either end of the AAm gel cylinder (dyed yellow), and 5 V DC is applied for 3 min (A1). The graphite anode (+) becomes strongly adhered to the gel (A2), allowing the pair to be lifted up in mid-air. Next, the graphite slabs are again contacted with the gel, and the polarity is reversed; i.e., the adhered slab is now the cathode (−). Upon applying 5 V DC for 3 min, the adhered slab is detached, while the bottom one (new anode) is now adhered to the gel. Scale bars: 1 cm. The entire experiment is also shown in Movie S1.

In this paper, we report our discovery that hard electronic conductors (e.g., metals or graphite) can be electroadhered to a range of soft aqueous materials including hydrogels, fruit, and animal tissue. Our studies into these hard–soft combinations arose as an offshoot of our earlier work on gels and tissues. The experiments are very simple. For example, two graphite slabs are placed on either side of a cylindrical hydrogel (5 cm tall), and 5 V DC is applied across the combination for ∼3 min. After this period, one of the graphite slabs is found to be strongly stuck to the hydrogel (see Figure 1). This adhesion endures long after the field is removed (gel-graphite pairs have remained adhered for months). We term this phenomenon as *hard–soft electroadhesion* or **EA**^[HS]^, and we emphasize that it is conceptually different from all previous uses of the term “electroadhesion”. Adhesion can be achieved in just a few seconds if the gel has high ionic conductivity. The adhesion is very strong: the adhesion strength is limited mostly by the strength of the gel and is shown to exceed 150 kPa.

Over the course of this study on **EA**^[HS]^, we have examined numerous hard–soft material pairs. On the soft side, **EA**^[HS]^ works with chemical gels like AAm, physical gels like gelatin and alginate, and even soft objects like fruit (bananas and apples) and animal tissue (beef, pork). Cationic, anionic, and nonionic gels can all be bonded to hard solids by this method. On the hard side, **EA**^[HS]^ is achieved with many metals (e.g., copper, lead, tin, nickel, iron, or zinc). Depending on the gel chemistry, adhesion occurs at the anode (+), cathode (−), both electrodes, or neither. If **EA**^[HS]^ is observed only to one electrode, generally it can be reversed by switching the polarity of the electrodes and reapplying the field. With regard to the mechanism behind **EA**^[HS]^, we show that it arises from electrochemical reactions that generate bonds between the hard electrode and the polymers in the gel network. Overall, this phenomenon is remarkable in its simplicity and wide applicability; in fact, one wonders why it has not been discovered earlier. We close with examples of hybrid materials created by **EA**^[HS]^ that highlight its utility in robotics, energy storage, biomedical implants, and surgery.

## Results and Discussion

### Reversible Adhesion of Graphite to AAm Gels

We first tested the adhesion between graphite and acrylamide (AAm) gels (Figure 1). The AAm gel was made by free-radical polymerization using 20% AAm, with *N*,*N*′-methylenebis(acrylamide) (BIS) (1.5% of the AAm) as the cross-linker. Salt (1% NaCl) was added to the gel for ionic conductivity. We typically prepared gels in the shape of a cylinder (2 cm diameter, 5 cm tall, total weight ∼30 g). This geometry allowed us to easily check if a hard solid was strongly adhered to the gel: as shown in the figure, when adhesion occurs, the solid is able to hold the gel in mid-air. The graphite slabs were cut from a larger piece to a size of 3 × 2 × 0.2 cm. As a control, when a graphite slab and the AAm gel were pressed into contact, there was no adhesion, and the two could be separated right away. Next graphite slabs are placed on the top and bottom of the gel (Figure 1A, Panel A1), and these are connected to a DC power supply. The graphite slabs thus serve as electrodes; i.e., one is the anode, connected to the positive terminal of the power supply, and the other is the cathode, connected to the negative terminal. With this setup, 5 V of DC is applied across the gel for 3 min. After the DC field is stopped, the graphite anode is found to be strongly adhered to the AAm gel. Panel A2 shows the anode lifting the gel in mid-air.

The results presented above were surprising and unexpected. Qualitatively, we noted right away that a strong adhesion had been induced between the gel and the slab. If we tried to wrench apart the gel and the slab, typically the gel would break, and pieces of the gel would be left behind on the graphite surface. The adhesion persisted indefinitely as long as the gel did not lose water (i.e., if the graphite-gel pair was stored in a closed container). We have preserved such adhered pairs in the lab for months, and they still remain adhered. If the gel is left to dry in air, it shrinks considerably, and then the adhesion to the slab weakens gradually due to a size mismatch.

A continuation of the experiment between graphite and AAm gel is shown in Figure 1B. We take the graphite-gel pair and place back the unadhered graphite slab on the other side. Then we apply the DC field in the reverse direction; i.e., we switch the polarity (Panel B1). The previously adhered graphite anode is now the cathode (−), while the other graphite slab serves as the new anode (+). With this configuration, we apply 5 V DC for 3 min (Figure 1B). After the DC field is stopped, the previously adhered graphite is found to have detached (Panel B2). Conversely, the previously unadhered slab is now stuck to the gel. These results imply that graphite adheres to AAm gels if it is the anode in a DC circuit but not if it is the cathode. Moreover, this implies that the adhesion can be reversed as and when desired by applying a DC field with reversed polarity. We have provided a movie (Movie S1) showing the entire experiment in Figure 1, including both graphite-AAm adhesion and its reversal.

### Factors that Affect the Adhesion Strength

**Figure 2:**
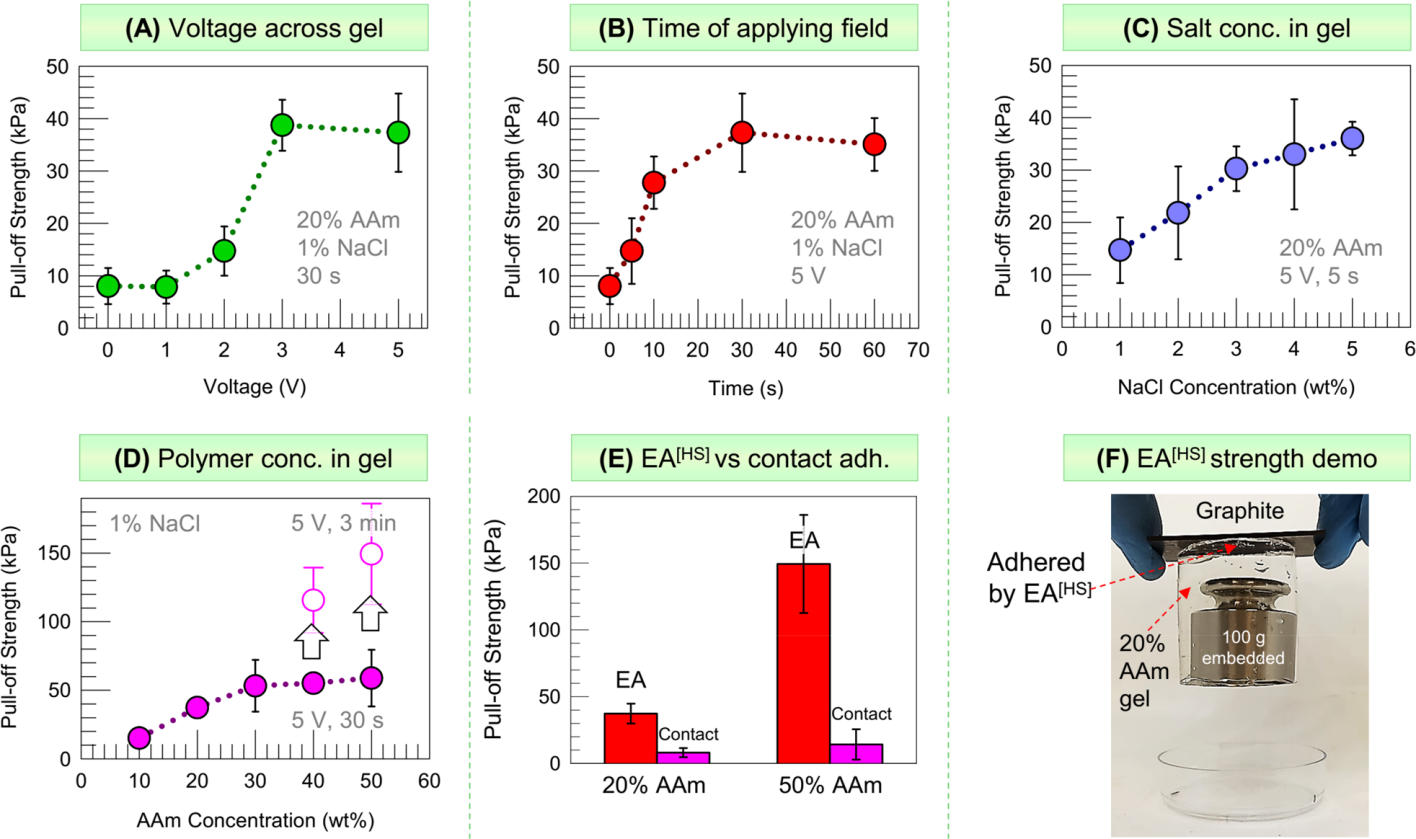
Factors that affect the adhesion strength achieved by **EA**^[HS]^ between graphite and AAm gels. The pull-off adhesion strength is shown in each graph. Mean values are plotted, and the error bars represent standard deviations from *n* ≥ 3 measurements. (A) Varying the voltage across the gel; (B) Varying the time over which the voltage is applied; (C) Varying the concentration of salt (NaCl) in the gel; (D) Varying the concentration of monomer (AAm) used to make the gel. The filled symbols correspond to 30 s of applying the voltage, and the open symbols correspond to 3 min. (E) The **EA**^[HS]^ values from (D) for 20 and 50% AAm are compared with the values for contact adhesion. **EA**^[HS]^ is much stronger. To illustrate the strength of **EA**^[HS]^, (F) shows that a graphite-gel pair (gel is 20% AAm) can support an additional weight of 100 g.

The phenomenon shown by Figure 1 is termed *hard–soft electroadhesion* or **EA**^[HS]^. What are the main factors that affect **EA**^[HS]^? To determine this, we conducted pull-off testing on adhered graphite-AAm pairs, and the results are presented in Figure 2. The gel pieces for these tests were made as cuboids with a base of 1 × 1 cm and a height of 1.6 cm. The test setup is shown in Figure S1A (see Experimental Section for details). From the experiments, we obtained the pull-off adhesion strength, which is the tensile stress required to separate the gel from the graphite slab (i.e., the stress at break).

First, we present the effect of varying the voltage on graphite-AAm **EA**^[HS]^ (Figure 2A). The DC voltage was varied from 0 to 5 V across the AAm gels. The gel composition was fixed at the one used in Figure 1, and, in each case, the voltage was applied for 30 s. Below 1 V, the adhesion strength is negligible (i.e., it is comparable to contact adhesion). At 2 V, **EA**^[HS]^ is noticeably stronger than contact adhesion. In this case, when the gel is pulled off from the graphite, an *adhesive failure* occurs;^33^ i.e., the gel and graphite separate at their interface (Figure S1B). This mode of failure was consistently observed when the adhesion strength was low (<20 kPa). When the voltage is increased to 3 V, the adhesion strength increases to 40 kPa. In this case, a *cohesive failure* occurs;^33^ i.e., the failure occurs in the middle of the gel (Figure S1C). Such failure was always observed when the adhesion strength was high (>30 kPa). It indicates that the adhesion is so strong that it exceeds the gel strength. Consequently, we find that the measured adhesion strength due to **EA**^[HS]^ levels out as the voltage increases above 3 V.

Next, we varied the time over which the electric field was applied (Figure 2B). The AAm gel was the same as above, and the voltage was fixed at 5 V. Over the first 30 s, the adhesion of the gel to graphite strengthens with increasing time, indicating that **EA**^[HS]^ accumulates as the voltage is applied. Thereafter, the adhesion plateaus. We again observed an adhesive failure for the initial points (<20 kPa in adhesion strength) and a cohesive failure for the subsequent ones. The results indicate that for a 1.6 cm-tall gel sample, strong adhesion can be achieved within a minute of applying the field. For the taller gels studied in Figure 1 (5 cm height), a longer time (∼3 min) was needed to achieve strong **EA**^[HS]^. This is why we applied the field for a time of 3 min in Figure 1.

The salt (electrolyte) concentration in the gel also plays an important role in **EA**^[HS]^ (Figure 2C). For a gel that is nonionic like AAm, in the absence of salt, the ionic conductivity is very low. It is only with the addition of salt that the gel becomes conductive. A conductive gel is needed to complete the DC circuit. As the salt in the gel increases, the current in the circuit increases. This has an effect on the adhesion strength, as shown in Figure 2C. For these experiments, we used the same 20% AAm gel and applied a voltage of 5 V for 5 s. NaCl was used as the salt, and its concentration in the gel was varied. The results show that the adhesion strength of the gel to graphite increases monotonically with increasing NaCl. A key corollary of this result is that if the gel has high salt, it can be stuck by **EA**^[HS]^ to hard solids in a very short time. For example, a tall (5 cm) AAm gel with 15% NaCl can be adhered strongly to graphite in only 5 s. We will return to this result in Figure 8.

We also studied the effect of the gel properties on **EA**^[HS]^. A first key variable is the concentration of polymer chains, which can be altered via the AAm monomer content used during synthesis. The gels so far all had 20% AAm with the cross-linker BIS at 1.5% of the AAm. We kept the same BIS:AAm ratio and varied the AAm from 10 to 50%. As the AAm increased, the gels transformed from soft to stiff (see the rheological data in Figure S2). We loaded each gel with 1% NaCl and studied their adhesion to graphite induced by a 5 V DC for 30 s. Figure 2D shows that the adhesion strength increases with the polymer concentration. One point to note here is that the values for the 10, 20, and 30% AAm gels correspond to the maximum value of adhesion strength at 5 V. That is, the time of 30 s was sufficient for these gels to reach a plateau in adhesion strength vs time (as found in Figure 2B). For the 40 and 50% AAm gels, increasing the time in the field beyond 30 s to 3 min increased the adhesion strength (open symbols in Figure 2D). For the 50% AAm gel, the bar graph in Figure 2E contrasts the **EA**^[HS]^ adhesion strength, which is ∼150 kPa, with the value for contact adhesion, which is ∼15 kPa. The comparison implies that **EA**^[HS]^ can be 10× the strength of contact adhesion if the gel is strong. Figure 2F shows a 100 g weight embedded in a 20% AAm gel and then stuck to graphite by **EA**^[HS]^. The pair can be held vertically, which vividly shows the high strength of **EA**^[HS]^.

### Adhesion to Various Hard Materials

**Figure 3:**
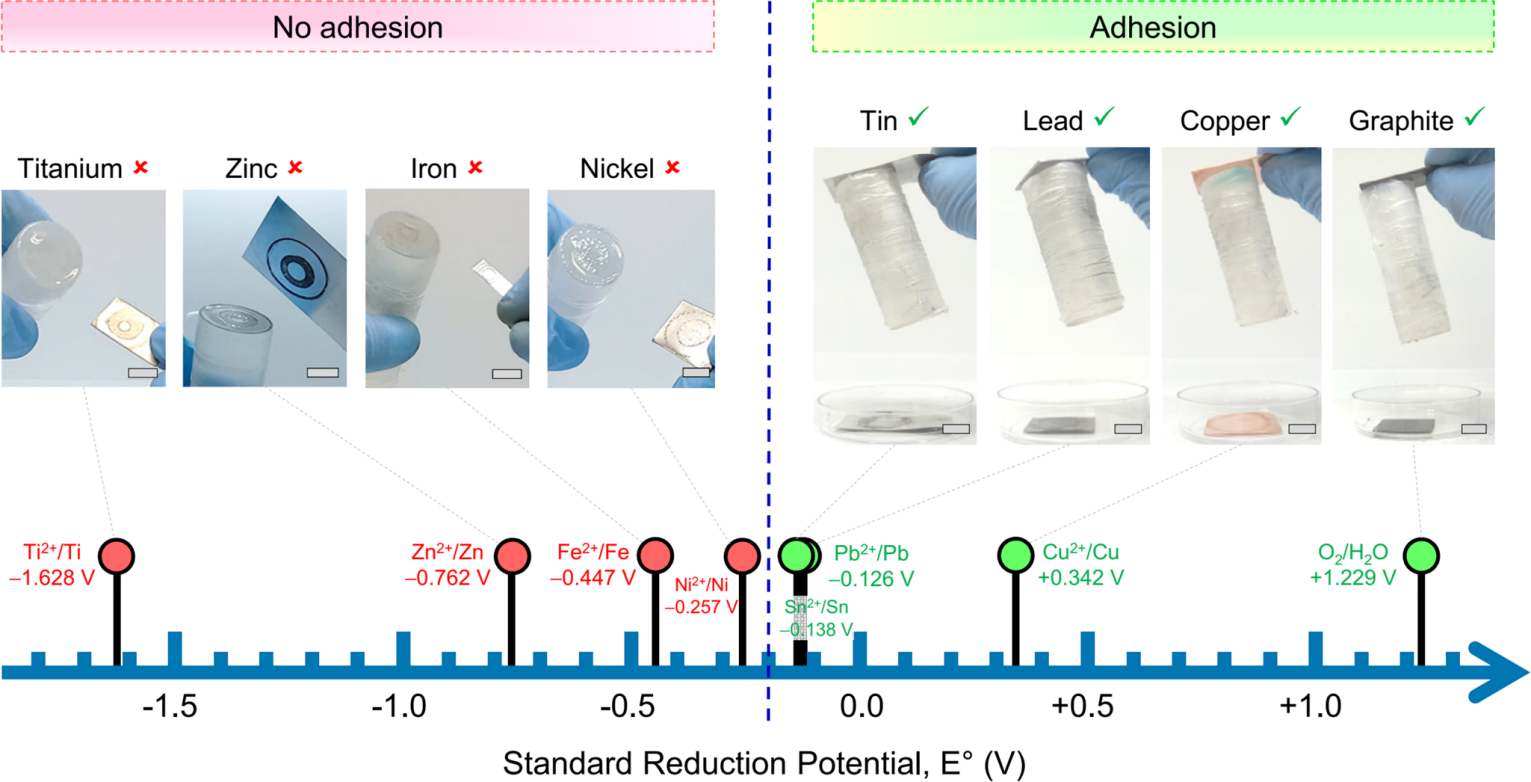
Adhesion results at the anode for various hard materials to AAm gels by **EA**^[HS]^. The results are shown in an electrochemical series with the standard reduction potential *E*° for each material. Photos are shown for each case. Strips of each material are placed on either end of a cylindrical AAm gel, and 5 V DC is applied for up to 15 min. Adhesion occurs only to the anode (+). Materials that adhere are all on the right side of the series, i.e., their *E*° > −0.2 V, indicating that they are relatively inert. In all these cases, the material-gel pair can be lifted up in the air. Materials that do not adhere have more negative *E*°, indicating that they are more reactive (easily oxidized). Scale bars are 1 cm.

Apart from graphite, what other hard materials can be stuck by **EA**^[HS]^ to gels? To examine this aspect, we performed tests with AAm gels and different metals (Figure 3). The gels are similar to those in Figure 1: 20% AAm with 1% NaCl and in the form of a 5 cm-tall cylinder. Each metal is in the form of rectangular strips (∼3 × 1 cm) with 0.2 to 0.8 mm thickness. The metal strips are placed on either side of a gel cylinder, and 5 V DC is applied for up to 15 min. All metals showed negligible contact adhesion to the gel (i.e., there was no adhesion in the absence of the field). Upon applying the field, **EA**^[HS]^ is induced with several metals on the anode (+) side, but there is no adhesion to the cathode (−) side. When adhesion occurs, the metal-gel pair can be lifted up in air, and this adhesion persists afterward.

The results for anodic adhesion to AAm are shown in Figure 3. Graphite, copper (Cu), lead (Pb), and tin (Sn) all adhere to AAm gels, while nickel (Ni), iron (Fe), zinc (Zn), and titanium (Ti) do not. We have arranged all the above in an electrochemical series,^34^ whereupon a pattern emerges. Metals that do not adhere to AAm have negative standard reduction potentials *E*°, indicating that they are more reactive;^34^ i.e., they easily lose electrons and thereby get oxidized. Conversely, materials that do adhere to AAm are *relatively inert*.^34^ These have positive (or not so negative) *E*°, with a cutoff value for adhesion being around −0.2 V. The correlation between adhesion and the electrochemical series suggests that **EA**^[HS]^ arises due to electrochemical reactions at the interface. At the anode, where the half-reaction is oxidative, the DC field causes the gel to react electrochemically with the inert metal (instead of electrolyzing the metal into cations). We should note that the result from Figure 3 is for AAm gels only. Some metals on the left of Figure 3 like Zn and Fe do undergo **EA**^[HS]^ to other gels, as will be discussed below.

### Adhesion to Various Hydrogels

Next, what other hydrogels can be stuck by **EA**^[HS]^ to hard solids? To study this, we tested graphite along with gels of different chemistries. Some were chemical gels made by free-radical polymerization (similar to AAm but with different monomers); the cross-links in these gels are covalent bonds. Others were physical gels, e.g., gels of polysaccharides or proteins where the cross-links are physical, noncovalent bonds (e.g., ionic or hydrogen-bonds). The geometry was the same as in Figure 1: each gel in the form of a 5 cm-tall cylinder, while graphite was in the form of thin slabs. 5–10 V DC was applied for up to 15 min, and adhesion was assessed visually as shown in Figures 1 and 3.

**Figure 4:**
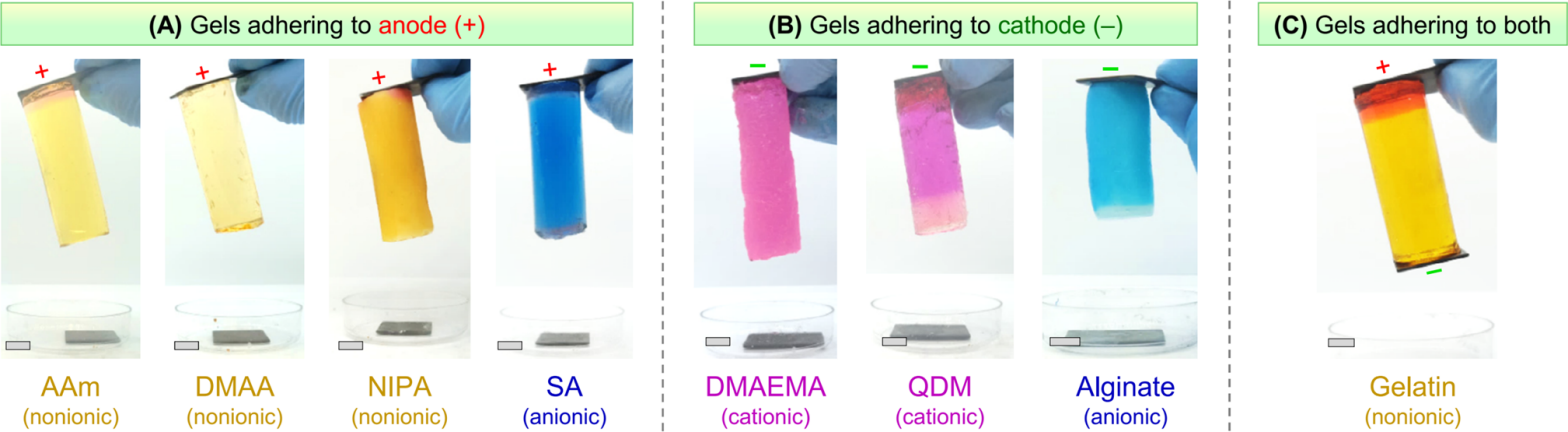
Adhesion results for graphite to various gels by **EA**^[HS]^. The results are shown through photos. Gels are imbued with dyes and are thus color-coded as follows: nonionic gels in yellow, anionic gels in blue, and cationic gels in pink. Strips of graphite are placed on either end of a cylindrical gel, and 5–10 V DC is applied for 15 min. (A) Gels that adhere only to the anode (+); (B) gels that adhere only to the cathode (−); (C) gelatin is the only gel that adheres to both electrodes. Scale bars are 1 cm.

The results for graphite-gel adhesion are presented in Figure 4. **EA**^[HS]^ is seen in many, but not all, cases, and the results are quite complex. We have color-coded the gels based on their ionic nature (nonionic, anionic, and cationic) using traces of water-soluble dyes. The same results, along with those from Figure 3, are also shown in tabular form in Table S1. First, the gels in Figure 4A all adhere to graphite only at the anode (+). These include AAm and other chemical gels made from the acrylic acid derivatives *N*,*N*-dimethylacrylamide (DMAA), *N*-isopropylacrylamide (NIPA), and sodium acrylate (SA).^2,3^ Note that AAm, DMAA, and NIPA gels are nonionic, whereas SA is anionic.

Next, the gels in Figure 4B all adhere to graphite only at the cathode (−). These include two cationic gels made by free-radical polymerization of the monomers [(2-methacryloyloxy)ethyl]trimethylammonium chloride (QDM) and 2-(dimethylamino)ethyl methacrylate (DMAEMA).^25,26^ Cathodic adhesion also occurs with gels of the anionic polysaccharide alginate, which is made by cross-linking sodium alginate with divalent calcium (Ca^2+^) cations.^26^ Thus, both cationic and anionic gels adhere to the cathodes. In the case of QDM gels, in addition to graphite, several metals (Cu, Pb, Sn, Ni, Fe, and Zn) all adhered at the cathode (Table S1). Note that this includes metals with both positive and negative reduction potentials.

Next comes the curious case of gelatin (Figure 4C). Gelatin is a denatured form of the protein collagen and forms thermoreversible gels in water driven by hydrogen-bonding of the protein chains into triple helices at cross-linking points.^4,5^ We find that gelatin undergoes **EA**^[HS]^ to graphite at *both the cathode and the anode*. This is the only gel in our studies that shows this behavior. Because gelatin adheres to both electrodes, we find that this adhesion cannot be reversed by reapplying the field with reversed polarity.

The last category of gels is those that do not stick to graphite at either the anode or the cathode. Gels in this category (photos not shown) include the nonionic chemical gel made from 2-hydroxyethyl methacrylate (HEMA) and two other nonionic physical gels: those of the synthetic polymer poly(vinyl alcohol) (PVA)^35^ and the polysaccharide agarose.^36^ The fact that some gels do not adhere to hard materials is an important point to note. This means that **EA**^[HS]^ is not due to a simple, universal reaction between water and any solid surface. It depends on the chemistry of both the gel and the hard material.

### Adhesion to Animal and Plant Tissues

Apart from hydrogels, are there other soft materials that can be adhered to hard materials by **EA**^[HS]^? We explored this point with a variety of animal and plant-based soft materials, especially those that are available as edible foods. Adhesion was attempted to graphite slabs using 5 V DC for up to 15 min. In the cases of fruit or vegetables, the sample was cut open and the graphite was contacted with the fleshy interior. (Note that the outer skins of many fruits are hydrophobic and may have negligible ionic conductivity.^37^)

**Figure 5:**
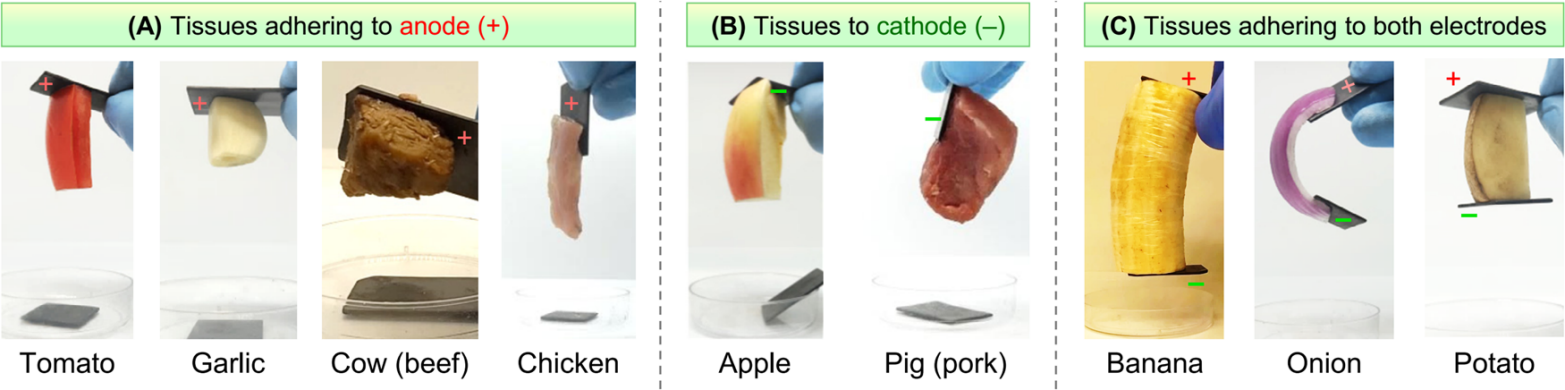
Adhesion results for graphite to various plant and animal tissues by **EA**^**[**HS**]**^. The results are shown through photos. Strips of graphite are placed on either end of a given soft material, and 5 V DC is applied for 15 min. (A) Tissues that adhere only to the anode (+); (B) tissues that adhere only to the cathode (−); (C) tissues that adhere to both electrodes.

The results on **EA**^[HS]^ with biological tissues are interesting, but again rather complex (Figure 5). Some tissues adhere to graphite only at the anode (+) (Figure 5A), and these include vegetables (tomato, garlic) as well as tissues from animals: cow muscle (beef shank) and chicken muscle (segment from the thigh). Some others adhere to graphite only at the cathode (−) (Figure 5B) and include fruit (apple) and pig muscle (pork shoulder). It is generally recognized that animal cells and tissues have an anionic character,^8,25^ but as can be seen here, the animal tissues we tested show wide differences in **EA**^[HS]^. Three of the plant materials we tested (banana, onion, and potato) adhere to both electrodes, similar to gelatin gels (Figure 5C). Lastly, there were several plant-based materials that did not adhere to either electrode, including grape, blueberry, raspberry, cucumber, orange, and pear (photos not shown).

The results in Figures 3–5 indicate that **EA**^[HS]^ works with a range of both hard and soft materials. The common requirement for the hard material is that it has to be *an electronic conductor*, which includes graphite and metals. As for the soft material, it has to be *an ionic conductor*, which means it must contain water and salt.

### Adhesion in Various Configurations

**Figure 6:**
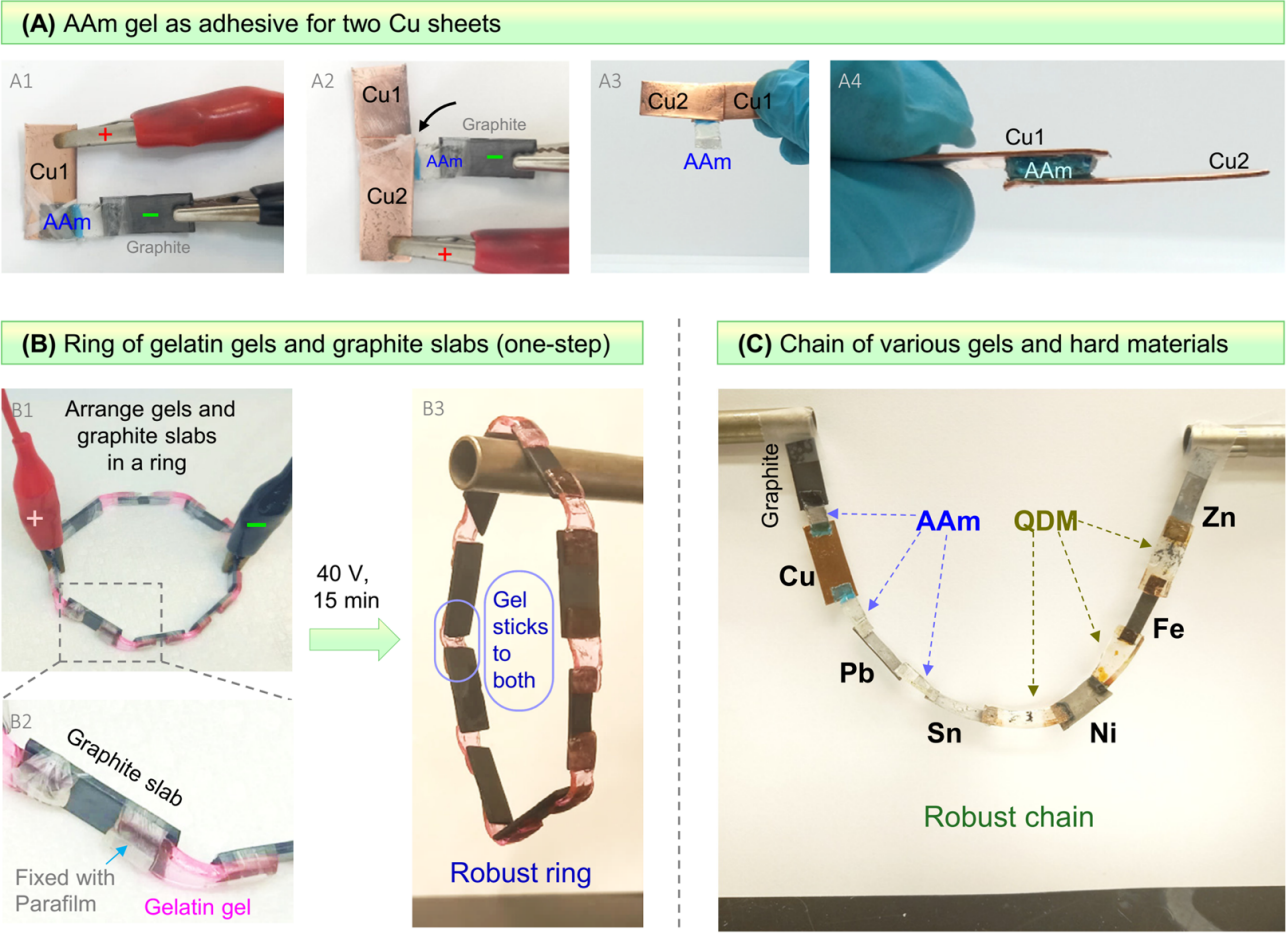
Use of **EA**^**[**HS**]**^ to adhere hard and soft materials in various configurations. (A) A thin AAm gel is used as an adhesive between two Cu sheets, labeled Cu1 and Cu2. AAm is first stuck to Cu1 using graphite as a counter electrode, and then the AAm is stuck to Cu2. (B) A ring of alternating gelatin gels and graphite strips is bonded together in a single step. This is possible because gelatin adheres to graphite at both electrodes. (C) A robust chain of eight different hard materials connected by AAm and QDM gels.

The versatility of **EA**^[HS]^ can be further shown by sticking hard and soft materials in other geometries or configurations, three of which are shown in Figure 6. First, we use a thin strip of AAm gel as an adhesive to stick two Cu sheets (Figure 6A). For this, an AAm gel (20% AAm, with 1% NaCl) is cut into a rectangular (3 × 1 cm) strip with a thickness of 2 mm. The Cu strips are also similarly sized (but thinner), as shown in Figure 6A, Panel A1. From Figure 3, we have seen that Cu strips adhere to AAm gels at the anode, whereas neither Cu nor graphite sticks to AAm at the cathode. With this knowledge at hand, we first place a Cu strip perpendicular to the AAm gel at one end and make this strip the anode (+) (Panel A1). At the other end of the gel, we place a graphite slab and make it the cathode (−). We then apply 5 V for 5 min, inducing **EA**^[HS]^ between the Cu anode and the gel. Then, a second Cu strip is placed over the gel (on the opposite side, parallel to the first Cu strip) and is made the anode (Panel A2). **EA**^[HS]^ between the second Cu and the gel is then induced (Panel A3). The overhanging portion of the gel is cut off, and we finally have the two Cu strips stuck together by the AAm gel that is sandwiched between them (Panel A4).

Another interesting demonstration is done with gelatin gels and graphite (Figure 6B). As noted earlier, gelatin gels adhere to graphite at both the anode and the cathode. We therefore attempt a single-step adhesion of gelatin and graphite pieces into a closed ring. For this, we begin with eight graphite slabs (3 × 1 × 0.2 cm size) and eight gelatin gel strips (3 × 1 × 0.2 cm size) and arrange them in a ring using Parafilm (Panel B1). Note that a portion of a given gel strip bridges adjacent graphite slabs (Panel B2). This is essentially a series configuration of hard and soft materials. We connect positive and negative terminals to two ends of the ring and apply a DC voltage of 40 V for 15 min. Each gel strip has one graphite slab connected to it as the anode and another as the cathode. Due to **EA**^[HS]^, all graphite-gel pairs adhere strongly, and the result is a robust ring. Panel B3 shows the ring being lifted up in air by a metal tube. This shows that the ring can be manipulated as a single object. The ring stays intact without a loss of adhesion indefinitely (as long as the gels remain hydrated).

A further possibility is to use **EA**^[HS]^ to adhere different gels and electrodes step by step, thereby generating unusual configurations such as a chain (Figure 6C). To make such a chain, a series of steps must be followed, and in each step, a given gel strip is contacted with a working electrode (WE) and a counter electrode (CE) and 10 V is applied for 2 min. The gel, the electrodes, and the direction of the electric field are chosen so that the gel only adheres to the WE but not the CE. Specifically, in the case of an AAm gel, it does not adhere to graphite at the cathode. Thus, a graphite cathode can be the CE, while Sn, Pb, Cu, and graphite can be the anode (WE). In the case of a QDM gel, it does not adhere to graphite at the anode. Therefore, a graphite anode can be the CE, while Sn, Ni, Fe, and Zn can be the cathode (WE). With these considerations, we start with a first gel strip, contact it with a WE and CE, and induce **EA**^[HS]^ between the gel and the WE. Next, this gel is contacted with a second WE and CE. By leaving the first adhered WE in an open circuit, we can adhere the second WE without affecting the already adhered pair. Thereby, a second (and different) metal is adhered to the gel strip on its other end. In this way, different gels and different electrodes are connected serially in a chain (Figure 6C). Graphite, Cu, Pb, and Sn are connected by AAm gel strips, while Sn, Ni, Fe, and Zn are connected by QDM gel strips.

### Mechanism for EA^[HS]^

We now turn to the crucial question regarding the mechanism: why does such adhesion occur? As discussed in the Introduction, **EA**^[HS]^ is conceptually distinct from all previous uses of the term “electroadhesion”. It arises between a hard electronic conductor and a soft ionic conductor. Once induced by the DC electric field, the adhesion persists thereafter. Adhesion is induced with both chemical and physical gels (Figure 4). Moreover, adhesion can be achieved to gels that are cationic, anionic, or nonionic. Some anionic gels such as SA stick to graphite only at the anode (Figure 4A), whereas other anionic gels such as alginate stick to graphite only at the cathode (Figure 4B). Thus, electrostatic or ionic interactions cannot be decisive factors in the mechanism behind **EA**^[HS]^.

Our results on **EA**^[HS]^ to AAm gels with different metals follow a significant trend (Figure 3). Metals adhere only at the anode to these gels, and those that do have positive reduction potentials, while those that do not have negative reduction potentials. This correlation with the electrochemical series strongly indicates that adhesion is caused by electrochemical reactions between the metal and the gel at the anode. Metals that do not adhere are those that getoxidized first at the anode. This oxidation (electrolysis) of the metal dominates over any competing processes, which explains why there is no adhesion. Conversely, metals that do adhere are relatively inert, allowing the polymer chains of the gel network to become oxidized first at the anode. We hypothesize that such oxidations result in chemical bonds between the metal surface and the polymer chains, leading to adhesion. The precise nature of the bonds will vary depending on the chemistry of the gel. When the polarity is inverted (i.e., the adhered surface is now made the cathode), the reactions at the electrode are reductive, which serves to undo the bonds between the metal surface and the polymer chains. As a result, the gel can now be detached from the metal.

**Figure 7:**
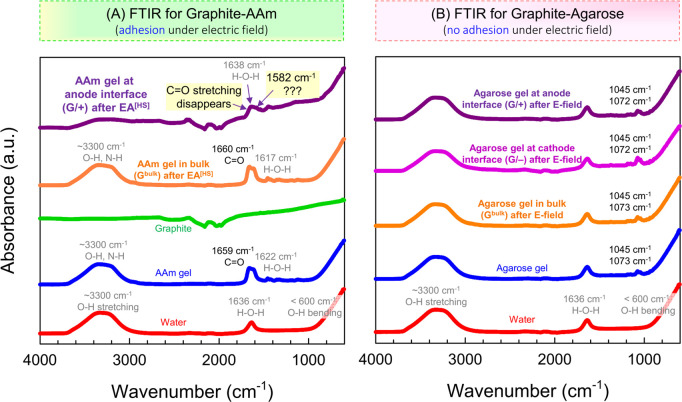
Probing the mechanism for **EA**^**[**HS**]**^ using FTIR. Spectra are shown for the cases of (A) graphite-AAm gel and (B) graphite-agarose gel. AAm adheres to graphite at the anode by EA^[HS]^, whereas agarose does not adhere to either electrode. Gel slices next to the electrodes or in the bulk are analyzed; see Figure S3 for details. No chemical changes are detected in (B) from the IR spectra. In (A), only the gel slice near the anode (denoted as G/+, purple curve) shows evidence of new bonds. A close-up of the key peaks is provided in Figure S4.

To test our hypothesis, Fourier transform infrared spectroscopy (FTIR) in the attenuated total reflectance (ATR) mode was conducted on the graphite-AAm pair (Figure 7A). The IR spectrum for a bulk AAm gel is dominated by the water present in it,^38,39^ as can be seen from the bottom two curves. Water shows a broad peak at 3300 cm^–1^ for O–H stretching, one peak at 1636 cm^–1^ for H–O–H scissoring, and one below 600 cm^–1^ for O–H bending. For the AAm gel, all these peaks appear, and there is an additional strong peak at 1659 cm^–1^ for the stretching of the C=O bond in the amide group.^38,39^ For a polished graphite surface without any contact with gels, IR absorption occurs over the range of wavelengths but with no clear peaks. Next, graphite electrodes were contacted with the AAm gel, and 5 V DC was applied for 15 min. As expected, the graphite anode adhered to the AAm gel by **EA**^[HS]^, while the cathode did not. We used a razor blade to cut slices of the gel next to each electrode as well as in the bulk (middle), i.e., far from the electrode interfaces. Photos of these slices are shown in Figure S3A and are labeled G/+, G/–, and G^bulk^.

Figure 7A shows that the AAm gel slice from the bulk (G^bulk^) has nearly the same IR spectrum as the one before adhesion (compare the blue and orange curves). This indicates that any changes to the gel happen only at the interfaces with the electrodes. Next, we turn to the gel slice next to the anode (G/+): note from the photos in Figure S3A that this gel still has some graphite attached to it, and this graphite cannot be washed off with water. The IR spectrum for G/+ is the top (purple) curve in Figure 7A and it is an overlap of the spectra for graphite and the gel. More importantly, the C=O stretching peak of the amide group has disappeared, while one additional peak now appears at 1582 cm^–1^. A close-up of these data showing the peaks is provided in Figure S4A. The data indicate that there must have been electrochemical reactions induced by the field that consume the amide group. In turn, these reactions appear to generate new bonds that are hard to identify. Conversely, the slice of gel next to the cathode (G/–, where there is no adhesion) shows an IR spectrum more similar to that of the bulk gel (Figure S4B). Incidentally, the photos in Figure S3A show this gel to also have some black graphite on it, but this graphite can be washed off easily, leaving a clear gel.

To further examine our hypothesis, we conducted FTIR on the graphite-agarose pair (Figure 7B). As shown in Figure 4D, this is a combination for which **EA**^[HS]^ does not occur. The initial agarose gel has water peaks as well as additional minor peaks at 1045 and 1073 cm^–1^. Next, the agarose gel was contacted with graphite and 5 V DC was applied for 15 min. A razor blade was then used to cut slices of the gel from the bulk (middle), the anode interface, and the cathode interface (see Figure S3B). Because there is no adhesion, the gel slices at the interfaces have no graphite clinging to them. IR spectra of the three gel slices (the top three curves in Figure 7B) are nearly identical and show no new peaks. This indicates that no electrochemical reactions have occurred at the interfaces (at least none that alter the chemistry of the gel).

We emphasize the contrast between Figure 7A,B. For graphite-AAm, where **EA**^[HS]^ occurs, IR shows *evidence of chemical changes to the gel at the adhering electrode* after the field is applied. For graphite-agarose, where no such adhesion occurs, there is no evidence of any chemical changes. This strengthens our hypothesis that adhesion arises due to electrochemical reactions between the adhering electrode and the gel. The precise nature of the bonds between the electrode and gel will depend on their chemistries.^12,21^ In the case of the new peak at 1582 cm^–1^ for AAm-graphite, we cannot precisely identify from IR databases what this peak represents. However, this region of the IR spectrum seems to correspond to alkenes or aromatic rings.^38^ In Table S2, we speculate on some possibilities for the bonds that may arise between graphite and AAm as well as a few other hard–soft pairs.

### Applications

**Figure 8:**
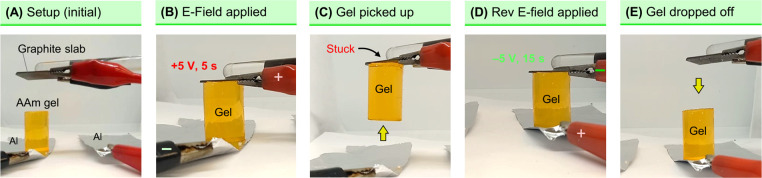
Electrogripper based on **EA**^**[**HS**]**^. Images are stills from Movie S2. (A) A graphite slab is stuck to a glass rod and connected to a DC power supply. Two pieces of Al foil serve as counter electrodes. An AAm gel to be picked up is placed on one foil. (B) The graphite slab is in contact with the gel and serves as the anode(+). 5 V is applied for 5 s. (C) The gel is stuck to the graphite slab and is picked up. (D) The gripper moves the gel to the other foil. The graphite is now made the cathode (−), and 5 V is applied for 15 s. (E) The gel detaches from the graphite and is dropped off.

We close this paper with a few demonstrations that leverage the use of **EA**^[HS]^. First, we show in Figure 8 an electrogripper to pick up and drop off gels (these are stills from Movie S2). We stuck a graphite slab to the end of a glass rod using epoxy glue and used this graphite as the working electrode (WE). Two pieces of aluminum (Al) foil serve as the counter electrodes. Two DC power sources (not shown in the figure) are used for adhesion and detachment, respectively. The gel cylinder (∼5 cm tall) is made with 20% AAm gel and contains 15% NaCl. The high salt ensures a high ionic conductivity and thereby a short adhesion time. Initially, the gel is placed on the first Al foil (Figure 8A). The graphite WE is placed in contact with the gel, and 5 V is applied for 5 s (Figure 8B): note that the graphite is the anode (+), while the Al is the cathode (−). Even with this short time, the graphite strongly adheres to the gel by **EA**^[HS]^, allowing the two to be lifted up in the air (Figure 8C). The gel-graphite pair is then placed on the second Al foil, and a reverse voltage of 5 V is applied for 15 s (Figure 8D), with the graphite as the cathode (−) and the Al as the anode (+). In this time, the graphite detaches from the gel and can be lifted off, leaving the gel on the Al foil (Figure 8E). In this way, the gel is picked up from one spot and dropped off at another. This setup could provide a simpler alternative for grippers in robotics,^27,30^ as it does not require the robot’s fingers or hands to have any joints or specific shapes to hold an object.

**Figure 9:**
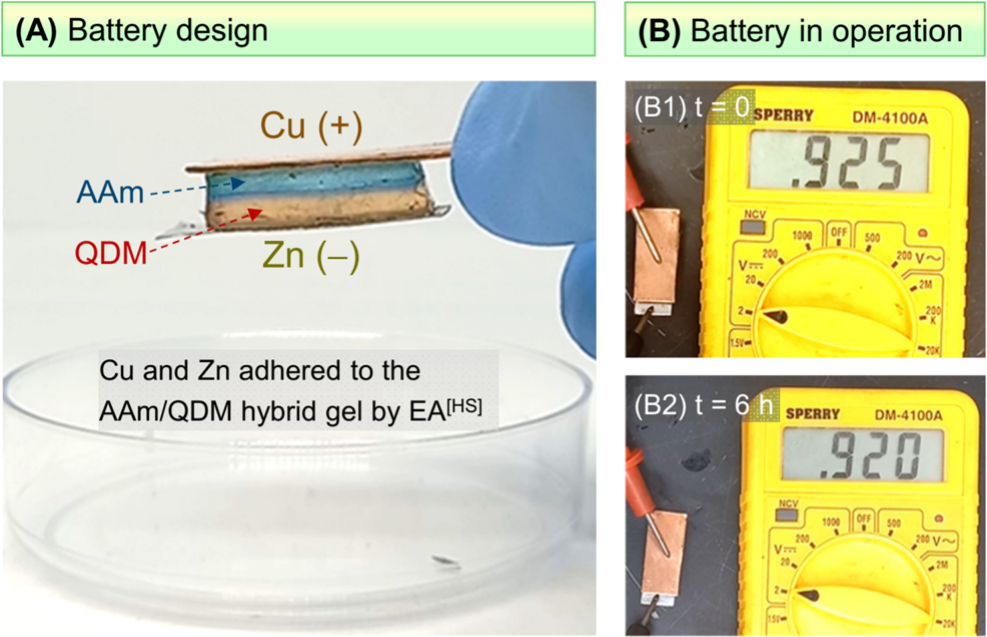
Primary battery created by **EA**^**[**HS**]**^. (A) The battery has Cu and Zn strips as electrodes flanking a hybrid AAm/QDM gel (with 1% NaCl in it) as the electrolyte. The metal strips are adhered to the gel using **EA**^[HS]^. (B) Under open-circuit conditions, with Cu as the cathode (+) and Zn as the anode (−), the battery delivers a potential of ∼0.9 V (B1), and this remains stable after 6 h (B2).

Another potential application of **EA**^[HS]^ is in making new kinds of batteries. Battery designs often have two hard solids (as electrodes) and an electrolyte between them. The electrolyte can be a soft solid, such as a gel in an ion-conductive solvent. We assembled a primary battery with a hydrogel electrolyte using our **EA**^[HS]^ technique (Figure 9A). The gel is a hybrid composed of two layers, with the top layer being AAm and the bottom being QDM. This hybrid gel was made using a strategy modified from our previous study^40^ (see Experimental Section for details). Cu and Zn were chosen as the electrodes. The logic behind these choices is that AAm adheres to Cu anodes by **EA**^[HS]^ (Figures 3, 6A), while QDM adheres to Zn cathodes by **EA**^[HS]^ (Figure 6C). We proceeded to place a Cu strip in contact with the AAm side and a Zn strip in contact with the QDM side. The gels both had 1% NaCl in them, as usual, for ionic conductivity. Then, with Cu as the anode and Zn as the cathode, we applied 10 V for 30 s. Both the metals adhered to the gels, as expected (Figure 9A). During this process, Cu was electrolyzed into Cu^2+^ within the AAm gel (note that the gel turns blue as a result). The overall assembly serves as a primary battery (Figure 9B). The open-circuit potential, with Cu as the cathode (+) and Zn as the anode (−), is ∼0.9 V. This output was stable for hours (Figure 9B), which shows that our simple setup provides the basic function of a battery. More sophisticated battery designs, including flexible and rechargeable batteries, can be assembled in the future using **EA**^[HS]^.

**Figure 10:**
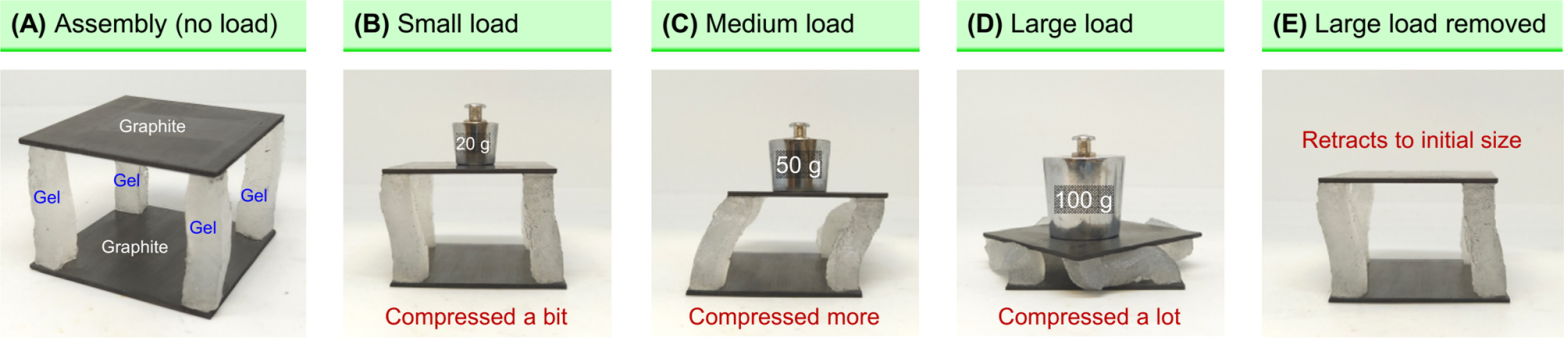
Load-bearing assembly fabricated by **EA**^**[**HS**]**^. (A) Four flexible gel pillars are electroadhered between two graphite slabs. Different weights are loaded on the top. (B) 20 g causes minimal compression. (C) 50 g causes the pillars to buckle and bend. (D) 100 g makes the pillars buckle until the top slab is compressed to the bottom one. (E) When the 100 g load is removed, the assembly retracts to its original state.

A third potential application of our **EA**^[HS]^ technique is in bioinspired actuators and soft robotics. By combining flexible hydrogels with rigid solid materials, we can fabricate robots with a stiff, bone-like skeleton as well as soft, muscle-like elements. Figure 10 shows a simple example of a load-bearing hard–soft structure made using **EA**^[HS]^. Two graphite slabs (5 × 5 cm) are joined by **EA**^[HS]^ to four pillars made of flexible AAm gels. To make the gels flexible, they are cross-linked by nanoparticles of the synthetic clay laponite, instead of the molecular cross-linker BIS.^40^ Each gel is made in the shape of a long cuboid (0.5 × 0.5 × 3 cm), and the gels are all robust and elastic. The gels are fixed as pillars on the four corners between the two slabs (Figure 10A). We then placed weights on the top slab to examine the ability of the structure to support a load. The structure is negligibly affected when 20 g is placed (Figure 10B), whereas 50 g makes the pillars buckle and bend (Figure 10C). When the load is increased to 100 g (Figure 10D), the gel pillars buckle, and the top slab is pushed down to the bottom one. When this weight is removed, the gel pillars retract to their original state, and so the top slab moves upward (Figure 10E).

The results in Figure 10 show the utility of combining hard and soft elements in the same structure. Hard elements alone will not be compressible or deformable, whereas soft elements alone could be crushed by a load. The combination, however, is able to bear a load without damage. Moreover, as the pillars retract after load removal (Figure 10E), the elastic energy stored in the deformed gels gets released, and in the process, the structure can do work (e.g., push an object). During all these steps, the *strong adhesion* induced by **EA**^[HS]^ between the hard slabs and the soft pillars persists and endures. This demonstrates that EA can indeed be leveraged in making actuators and robots. Similar hard–soft assemblies could also be useful in the body where metal implants like stainless steel or titanium are widely used. The ability to adhere a gel (or tissue) to metal could be useful in reinforcing these implants and also to control the interface between the implant and bodily fluids.

**Figure 11:**
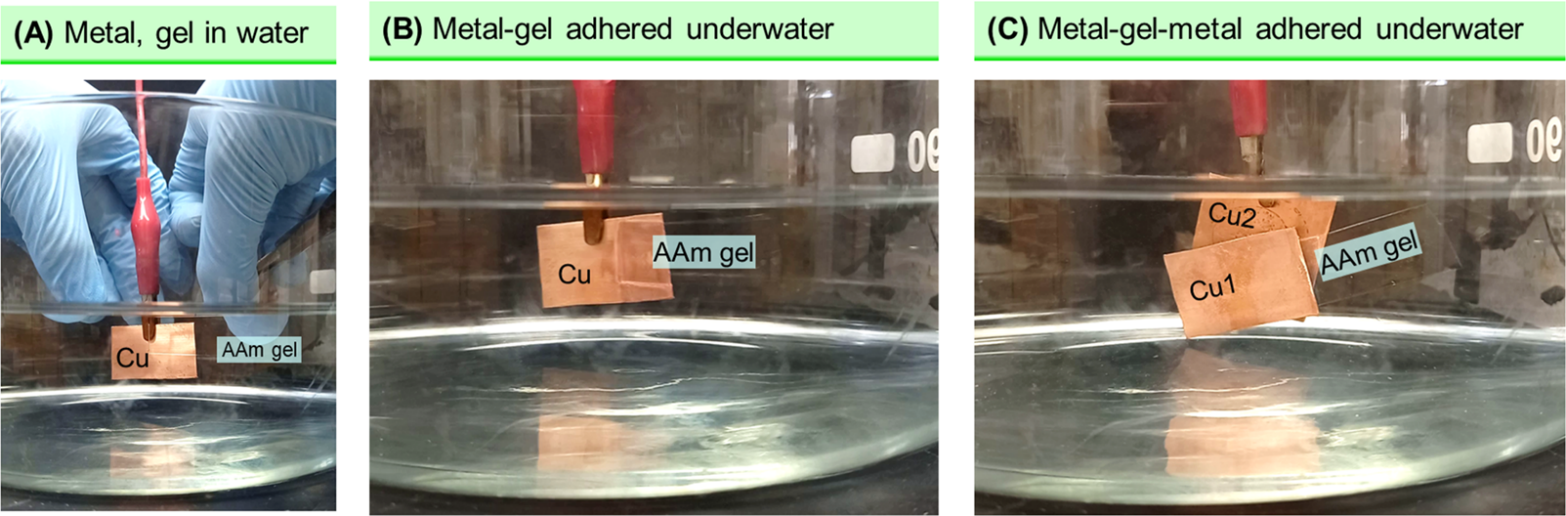
Underwater adhesion of metal and gel by **EA**^**[**HS**]**^. (A) A Cu sheet and an AAm gel are contacted underwater. With the Cu as anode and graphite as the cathode (not shown), **EA**^[HS]^ is induced between the metal and the gel. (B) After the field is switched off, the pair remain adhered. (C) Next, a second Cu sheet is adhered on the opposite side of the gel by **EA**^[HS]^. The gel thus serves as an underwater adhesive between the two metal sheets.

A final point is that the adhesion induced by **EA**^[HS]^ between hard and soft solids can also be achieved underwater. This is demonstrated in Figure 11 using Cu sheets and an AAm gel. First, in Figure 11A, we bring a Cu sheet into contact with a strip of AAm gel while immersed in water. We make Cu the anode (+) and complete the circuit with graphite as the cathode (−), similar to the arrangement in Figure 6A. The gel is the same as that in Figure 8 and has a high salt content (15% NaCl), which ensures a short adhesion time. We then apply 5 V for 60 s, thus inducing **EA**^[HS]^ between the metal and the gel (Figure 11B). Note that the graphite as cathode does not adhere to the gel and is not shown in the figure. Next, we stick a second Cu sheet to the opposite side of the AAm gel strip. For this, the second Cu sheet is made the anode, graphite is again the cathode, and the adhered Cu sheet is left in an open circuit. 5 V is again applied for 60 s to induce **EA**^[HS]^. The result (Figure 11C) is that the two Cu sheets are stuck together by the AAm gel, similar to the earlier result in Figure 6A. However, in the present case, the entire assembly is underwater; thus, *the gel is able to serve as an underwater adhesive*. Achieving adhesion underwater has proven to be a huge challenge in recent years because many flowable adhesives cannot be spread onto solid surfaces that are immersed in liquids like water. Even if spreading can be achieved, the solid–solid adhesion ends up being quite weak because the bonds between the solids are influenced by the water molecules around them. Here, we are able to surmount this problem because the gel is not inherently adhesive to the metal: the adhesion is only switched on when the gel and metal are contacted under the field.

## Conclusions

In summary, we have reported a simple method for adhering hard materials to soft aqueous materials. The hard material must be an electronic conductor such as a metal or graphite, allowing it to serve as electrodes in a simple electrochemical setup. The soft material must be an ionic conductor, which typically means that it must have water and ions (salt). Examples of such materials include hydrogels, as well as plant-based tissues (fruits and vegetables) and animal-based tissues (meat from cows, pigs, and chickens). Our method, which we term *hard–soft electroadhesion* or **EA**^[HS]^, is to bring the hard and soft material into contact and apply a low DC electric field (e.g., 5 V) for a short time (e.g., 3 min). Depending on the nature of the hard and soft materials, adhesion is induced at the anode (+), cathode (−), both electrodes, or neither. This adhesion endures after the field is removed. If adhesion is observed for only one electrode, switching the polarity of the field typically reverses the adhesion. The adhesion strength increases with increasing voltage, time in the field, and the ionic conductivity of the gel. The ultimate adhesion strength is limited only by the strength of the gel. Metals that can be adhered via **EA**^[HS]^ to AAm gels (all at the anode) have reduction potentials above a critical value. This correlation with the electrochemical series suggests that **EA**^[HS]^ is due to chemical bonds between the gel and the anode induced by electrochemical reactions. Support for this conclusion comes from FTIR data. Finally, the versatility of this phenomenon is shown through various examples. **EA**^[HS]^ could enable applications in robotics, energy storage, and biomedical implants.

## Experimental Section

### Materials

The following monomers were from Sigma-Aldrich: acrylamide (AAm), *N*,*N*-dimethylacrylamide (DMAA), *N*-isopropylacrylamide (NIPA), 2-hydroxyethyl methacrylate (HEMA), 2-(dimethylamino)ethyl methacrylate (DMAEMA), sodium acrylate (SA), [2-(methacryloyloxy)ethyl]trimethylammonium chloride (QDM, 75% solution in H_2_O), and *N*,*N*′-methylenebis(acrylamide) (BIS). Other chemicals used for making hydrogels were also from Sigma-Aldrich, including the initiators ammonium persulfate (APS) and potassium persulfate (KPS) and the accelerant *N*,*N*,*N*′,*N*′-tetramethylethylenediamine (TEMED). Polymers used in this study were also from Sigma-Aldrich and included alginate (i.e., alginic acid sodium salt, from brown algae, medium viscosity), agarose (Type IA, low EEO), gelatin (from porcine skin, gel strength 300, Type A), and poly(vinyl alcohol) (PVA, MW 85–124k, 99+% hydrolyzed). Other chemicals included calcium chloride dihydrate (CaCl_2_, from Sigma-Aldrich), sodium chloride (NaCl, from LabChem), and hydrochloric acid (HCl, from BDH). Graphite sheets (∼1.5 mm thickness) were from Saturn Industries. Dyes used to color-code the gels were methyl orange from TCI America, and methylene blue and rhodamine B from Sigma-Aldrich. Laponite XLG was a gift from Southern Clay Products. Cu, Pb, Sn, Ni, Fe, Zn, and Ti were purchased from RotoMetals. All the meat, fruits, and vegetables were purchased from Whole Foods. Deionized (DI) water was used in all of the experiments.

### Hydrogel Synthesis

AAm, SA, DMAA, NIPA, QDM, DMAEMA, and HEMA gels were prepared by free-radical polymerization. For a typical gel, 20% monomer, 0.03–0.06% BIS (cross-linker), and 2.0 μL/g TEMED (accelerator) were dissolved in DI water. After sufficient mixing, 0.02–0.06% initiator (APS or KPS) was quickly mixed into the solution. Then the solution was placed under a nitrogen atmosphere for at least 30 min, whereupon it polymerized into a gel. For laponite-cross-linked AAm gels, 3% laponite XLG particles were used as cross-linker instead of BIS. Gels were typically made in 30 mL cylindrical vials, and when taken out of the vial, they had a cylindrical form with a size close to the vial dimensions, i.e., a height of 5 cm and a diameter of 2 cm. Such cylindrical gels were used in the adhesion studies shown in Figures 1, 3, and 4. For other experiments, the same gels were also made in Petri dishes. For all electroadhesion experiments, it was necessary to include salt in the gel to ensure the ionic conductivity. For this purpose, 1% NaCl was typically added to the monomer solution prior to polymerization.

Alginate gels were made by a new method that involves diffusion of Ca^2+^ cations; 3% alginate was dissolved in DI water in a vial. Then the solution was frozen at −20 °C overnight. The vial was then broken, and the frozen solid was removed. This cylindrical solid (∼2 cm diameter and 5 cm height) was then placed in a 7% CaCl_2_ solution at room temperature on a stir plate. As the solid melted, Ca^2+^ came into contact at the interface, and the cations diffused inward to cross-link the alginate chains. Within 6 h, an alginate gel in the shape of a cylinder was obtained, and this was used in Figure 4.

PVA gels were made by freeze–thaw cycling.^35^ 20% PVA (with 1% NaCl for ionic conductivity) was dissolved in DI water at ∼90 °C. Upon cooling to room temperature, a viscous solution was obtained. This solution was then subjected to two freeze–thaw cycles. For each cycle, the solution was frozen at −20 °C overnight and then thawed at room temperature. The freeze–thaw cycling induced the PVA to form a robust gel due to the formation of crystallites at junctions between the chains.^35^

Gelatin gels were made by dissolving 20% gelatin together with 1% NaCl in DI water at 50 °C. Upon cooling to room temperature, the solution was converted into a gel due to the formation of triple-helical junctions between the gelatin chains.^4,5^ Similarly, agarose gels were made by heating 5% agarose together with 1% NaCl in DI water at 90 °C. Upon cooling to room temperature, the agarose chains bind to each other via hydrogen-bonds, resulting in a gel.^36^

The hybrid AAm/QDM gel was made using a method modified from our previous work.^40^ The QDM gel was first made in a 90 × 10 mm Petri dish, as described above. After the QDM gelled, a pregel solution of AAm was added onto the top. Then the sample was placed again under a nitrogen atmosphere until the AAm also gelled. The AAm and QDM layers were strongly bonded with each other since the pregel solution would diffuse into the QDM gel, making the resulting AAm gel network penetrate with the existing QDM network. Then the hybrid gel was cut into an appropriate shape with a razor blade for adhesion experiments.

### Electroadhesion Experiments

Two polished slabs or strips of the hard material (graphite or metal) were placed in contact on either end of the gel (or other soft material) to be tested, as shown in Figure 1. The two slabs were connected as electrodes to the positive and negative terminals of a BK Precision 9104 DC power supply. A DC voltage of typically 5 to 10 V was applied for 3 to 15 min. If the duration of exposure to the electric field was relatively long, the gels were covered with Parafilm during the experiment to minimize water evaporation. After the field was stopped, the electrodes were examined for adhesion to the gel. Next, the polarity was reversed (i.e., the previous anode became the cathode, and vice versa), and the field was reapplied. The electrodes were then examined again for adhesion to the gel.

### Pull-Off Adhesion Strength Tests

The data shown in Figure 2 are for the strength of **EA**^[HS]^ between the AAm gels and graphite. For these measurements, AAm gels were made in Petri dishes, from which they were cut into cuboids with a square cross-section (1 × 1 cm) and a height of 1 to 1.6 cm. The gel was then electroadhered to a graphite slab (2 × 1.5 × 0.2 cm). After adhesion, the gel was cut open using a razor blade with ∼0.2 cm thickness left on the graphite slab. This gel-graphite pair was then taken for pull-off testing on an AR2000 rheometer (TA Instruments) using 40 mm parallel plates. As shown in Figure S1A, the back side of the graphite was stuck to the bottom plate using either double-sided tape or epoxy glue. On the top plate, a zinc sheet was first affixed using double-sided tape or epoxy glue. Then, the gel was stuck to the sheet using a cyanoacrylate glue. With this setup, the pull-off test mode was selected on the rheometer software. During the test, the top plate was pulled upward at a constant rate (typically 1.0–9.3 μm/s; the stiffer the gel, the lower the rate), and the normal-force transducer was used to record the normal force as a function of the gap (distance). The force was converted to stress by dividing by the contact area between the gel and the graphite. The stress at the point of failure was taken as the pull-off strength for a given gel-graphite pair.

### Rheology

Rheological experiments on the AAm gels (Figure S2) were performed at 25 °C on an AR2000 stress-controlled rheometer (TA Instruments) using 20 mm parallel plates. Gels were cut into discs of diameter 20 mm and thickness 2 mm. Dynamic stress-sweeps were first performed to identify the linear viscoelastic (LVE) region of the sample. Dynamic frequency sweeps were then conducted at a constant strain amplitude within the LVE region.

### Infrared Spectra

Infrared spectra were collected using a Fourier transform infrared (FTIR) spectrometer (Thermo Nicolet NEXUS 670) in the attenuated total reflectance (ATR) mode. Samples examined were typically those at gel-electrode interfaces (see Figure S3). Samples were directly placed on the ATR window in the instrument, and absorption spectra were measured over a wavenumber range of 650–4000 cm^–1^ with a resolution of 4 cm^–1^. Each sample underwent 32 scans, and the resulting spectra was averaged.

### Statistics

For the data in Figure 2, at least three samples were tested for each data point. No outliers were excluded. Mean values are shown in the plots, and error bars correspond to standard deviations. Statistics were calculated and plotted by using Excel and SigmaPlot.

## Supporting Information Available

Figure S1: Pull-off testing and failure modes. Figure S2: Rheology of AAm gels studied for **EA**^[HS]^ to graphite. Figure S3: Gel samples prepared for analysis by FTIR. Figure S4: Additional FTIR data and analysis. Table S1: Results for **EA**^[HS]^ with various hard and soft materials. Table S2: Bonds induced between gels and graphite by **EA**^[HS]^. In addition, the SI contains Movies S1 and S2, which accompany Figures 1 and 8 in the text, respectively (PDF)
Movie S1: Reversible adhesion of graphite to AAm gel by **EA**^[HS]^ (MP4)
Movie S2: Electrogripper based on EA^[HS]^ (MP4)
Transparent Peer Review report available (PDF)
