# Supplementary material for: Reversibly Sticking Metals and Graphite to Hydrogels and Tissues
Source: ACS Cent Sci. 2024 Mar 13;10(3):695–707. doi: 10.1021/acscentsci.3c01593 (PMC10979492; doi:10.1021/acscentsci.3c01593)

Supporting Information for:

Reversibly Sticking Metals and Graphite to Hydrogels and Tissues

Wenhao Xu, Faraz A. Burni and Srinivasa R. Raghavan*

Department of Chemistry & Biochemistry
Department of Chemical & Biomolecular Engineering
University of Maryland
College Park, Maryland 20742, USA

*Corresponding author. Email: sraghava@umd.edu

Contents

Figure S1: Pull-off testing and failure modes.

Figure S2: Rheology of AAm gels studied for their adhesion by EA[HS] to graphite.

Figure S3: Gel samples prepared for analysis by FTIR.

Figure S4: Additional FTIR data and analysis for graphite-AAm adhesion.

Table S1: Results for EA[HS] with various combinations of hard and soft materials.

Table S2: Hypothesiszed bonds induced between different gels and graphite by EA[HS].

Movie S1: Reversible adhesion of graphite to an AAm gel by EA[HS].

Movie S2: Electrogripper based on EA[HS].

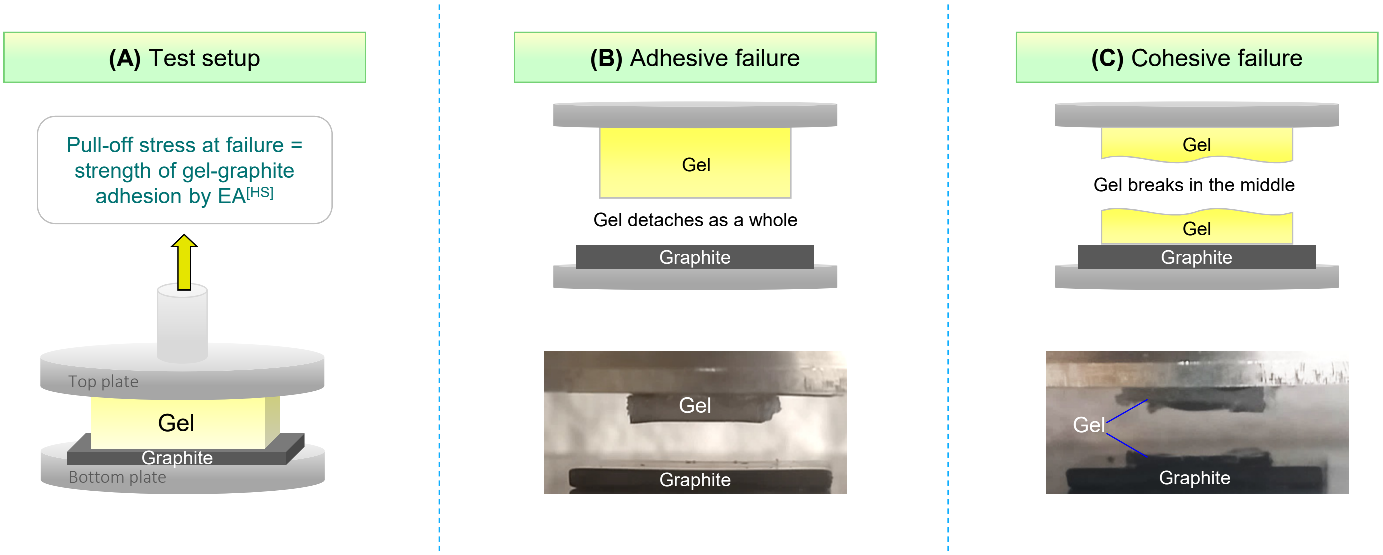

Figure S1. Pull-off testing and failure modes. (A) A schematic of the pull-off testing setup is shown. Tests are conducted using a rheometer in a parallel-plate geometry. A given gel and graphite are adhered by EA[HS]. The gel is then glued onto the top plate and the graphite is glued onto the bottom plate. The top plate is then pulled upwards while the stress is monitored. The stress at the point of failure is the adhesion strength induced by EA[HS]. (B) When the adhesion strength is low (below ~ 20 kPa), the failure mode is *adhesive*, i.e., the gel neatly detaches as a whole from the graphite. This is shown by the photo and the schematic. (C) When the adhesion strength is high (above ~ 30 kPa), the failure mode is *cohesive*, i.e., the gel breaks in the middle. This is shown by the photo as well as the schematic. Cohesive failure indicates that the adhesion is stronger than the strength of the gel.

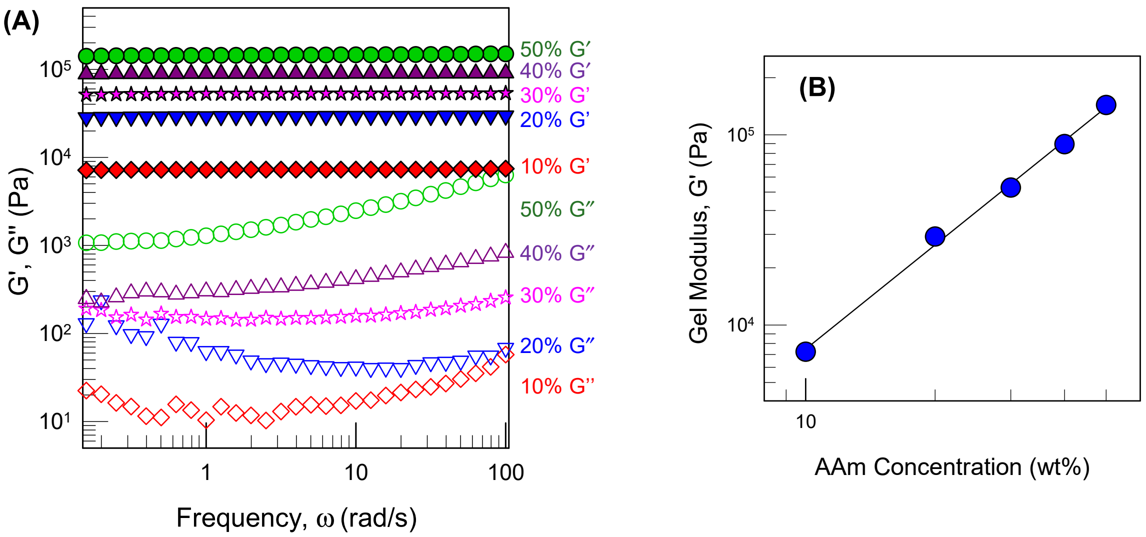

Figure S2. Rheological properties of acrylamide (AAm) gels studied with regard to their adhesion to graphite via EA[HS] in Figure 2D. (A) Data from dynamic rheology for the elastic modulus G' and the viscous modulus G'' as functions of the angular frequency ω. All samples show the rheology characteristic of a gel, with G' being independent of ω. Each gel can thus be characterized by its value of G', which is the gel modulus. (B) Plot of the gel modulus vs. AAm concentration, showing a power-law relationship. At low AAm (10%), the gel is soft (modulus ~ 7 kPa), whereas at high AAm (50%), the gel is very stiff (modulus ~ 150 kPa).

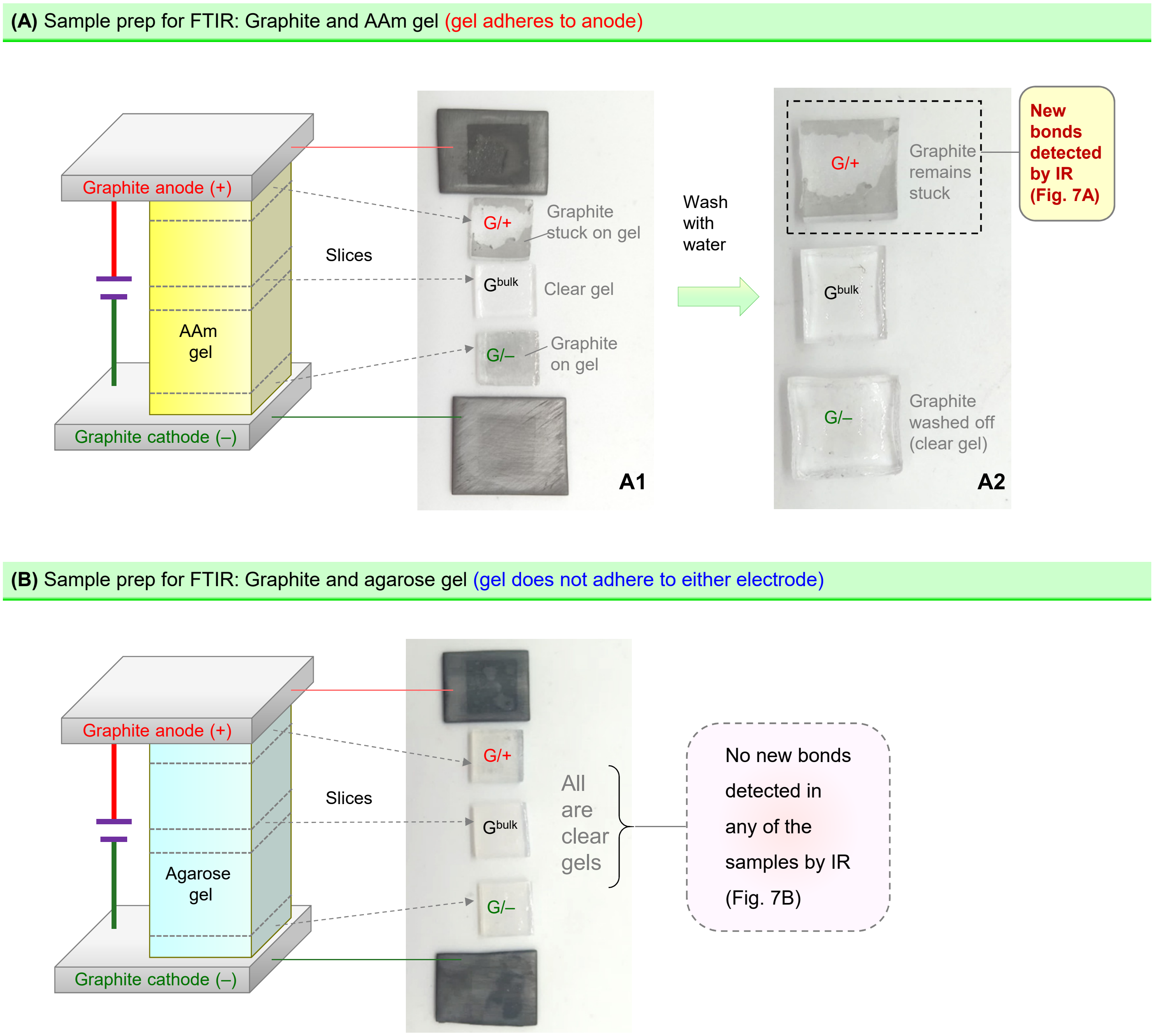

Figure S3. Gel samples prepared for analysis by FTIR. A tall cuboidal gel is contacted with graphite slabs on either end and 5 V DC is applied for 5 min. Next, thin slices of the gel are made at different locations. The slice in the middle is denoted as G^{bulk}. The slice next to the anode is G/+ and that next to the cathode is G/–. These gel slices are then analyzed by FTIR-ATR. Two gels are considered. (A) A gel of acrylamide (AAm), which adheres by EA[HS] to the anode. In this case, the anode slice (G/+) has some graphite clinging to it (Photo A1) and this graphite cannot be removed by washing with water (Photo A2). The cathode slice (G/–) also initially appears to have some graphite stuck to it (Photo A1), but this graphite is easily washed away with water. The G^{bulk} slice is clear. In FTIR (see Fig. 7A in the main paper), only G/+ shows evidence of new bonds. (B) A gel of agarose, which does not adhere to either electrode. In this case, all the gel slices are clear, and no new bonds are detected in any of them by FTIR (see Fig. 7B).

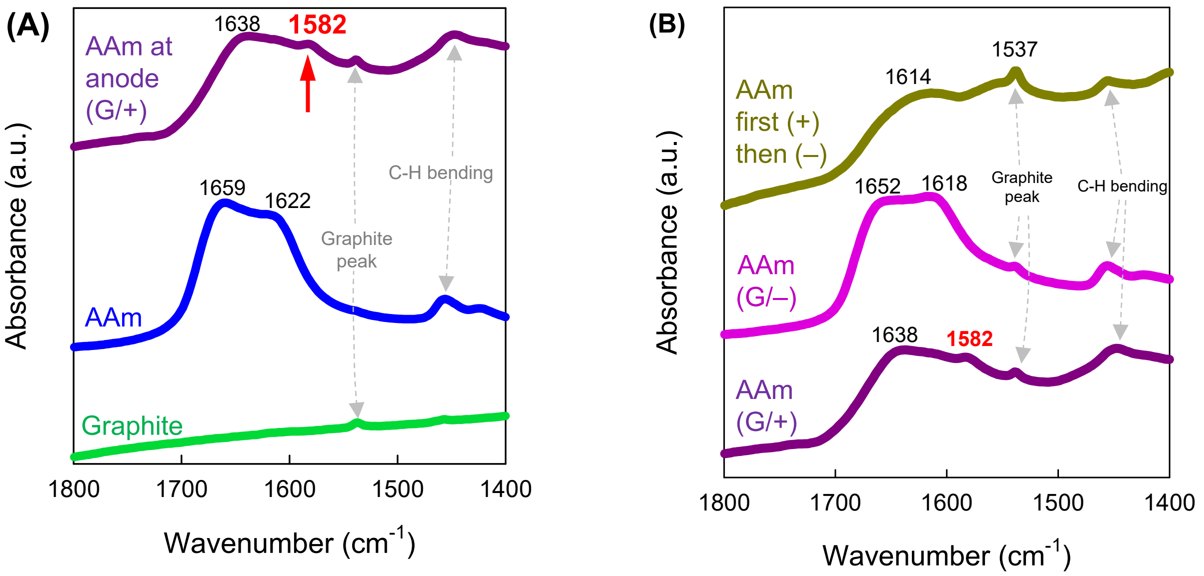

Figure S4. Additional FTIR data and analysis for the graphite-AAm gel pair. (A) Close-up of data shown in Figure 7A (main paper), highlighting the peaks in the vicinity of 1600 cm^{-1}. For the AAm gel slice near the anode (G/+), the new peak at 1582 cm^{-1} indicates new bonds induced by EA[HS] between graphite and AAm. (B) Data for the G/+ gel slice is compared to two other cases. First, the gel slice near the cathode (G/–) has a spectrum that is closer to the AAm in the bulk. Next, an AAm gel was adhered at the anode, then the polarity was reversed to detach the gel. The slice at this electrode interface is the top curve and it shows a more complex spectrum. Importantly, the peak at 1582 cm^{-1} is no longer present.

Table S1. Results from EA[HS] studies with various combinations of hard and soft materials. The results include those shown in Figures 3 and 4 in the main paper.
(A) Cases where the soft material is a nonionic AAm gel and the hard material is various metals. These are discussed under Figure 3.
(B) Cases where the hard material is graphite and the soft material is various gels. These are discussed under Figure 4.
(C) Cases where the soft material is a cationic QDM gel and the hard material is various metals.

A. Adhesion of AAm gels to various metals

Hydrogel material	Electrode material	Anodic (+) adhesion	Reversal of adhesion	Cathodic (–) adhesion	Reversal of adhesion
AAm	Graphite (C)	✓	✓	✗	-
AAm	Copper (Cu)	✓	✗	✗	-
AAm	Lead (Pb)	✓	✗	✗	-
AAm	Tin (Sn)	✓	✓	✗	-
AAm	Nickel (Ni)	✗	-	✗	-
AAm	Iron (Fe)	✗	-	✗	-
AAm	Zinc (Zn)	✗	-	✗	-
AAm	Titanium (Ti)	✗	-	✗	-

Graphite, Copper, Lead, Tin: Adhesion to inert metals at the anode
Nickel, Iron, Zinc, Titanium: No adhesion to reactive metals

B. Adhesion of graphite to various gels

Hydrogel material	Electrode material	Anodic (+) adhesion	Reversal of adhesion	Cathodic (–) adhesion	Reversal of adhesion
AAm (nonionic)	Graphite (C)	✓	✓	✗	-
DMAA (nonionic)	Graphite (C)	✓	✓	✗	-
NIPA (nonionic)	Graphite (C)	✓	✓	✗	-
SA (anionic)	Graphite (C)	✓	✓	✗	-
DMAEMA (cationic)	Graphite (C)	✗	-	✓	✓
QDM (cationic)	Graphite (C)	✗	-	✓	✓
Alginate (anionic)	Graphite (C)	✗	-	✓	✓
Gelatin (nonionic)	Graphite (C)	✓	✗	✓	✗
HEMA (nonionic)	Graphite (C)	✗	-	✗	-
Agarose (nonionic)	Graphite (C)	✗	-	✗	-
PVA (nonionic)	Graphite (C)	✗	-	✗	-

AAm, DMAA, NIPA, SA: Adhesion only to anode, reversible
DMAEMA, QDM, Alginate: Adhesion only to cathode, reversible
Gelatin: Adhesion to both
HEMA, Agarose, PVA: No adhesion

C. Adhesion of QDM gels to various metals

Hydrogel material	Electrode material	Anodic (+) adhesion	Reversal of adhesion	Cathodic (–) adhesion	Reversal of adhesion
QDM	Graphite (C)	✗	-	✓	✓
QDM	Copper (Cu)	✗	-	✓	✗
QDM	Lead (Pb)	✗	-	✓	✗
QDM	Tin (Sn)	✗	-	✓	✗
QDM	Nickel (Ni)	✗	-	✓	✗
QDM	Zinc (Zn)	✗	-	✓	✗
QDM	Iron (Fe)	✗	-	✓	✗

All rows: All adhere to cathode only

Table S2. Hypothesiszed bonds induced between different gels and graphite by EA[HS]. A schematic for the adhesion of an AAm gel to graphite via the pertinent bonds is shown below the table.

Hydrogel	Electrode	Oxidation/ Reduction	Hypothesized Bonds
AAm	Graphite (+)	Oxidation	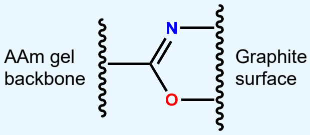
SA	Graphite (+)	Oxidation	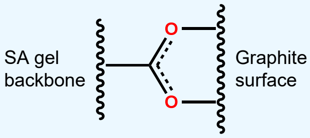
QDM	Graphite (–)	Reduction	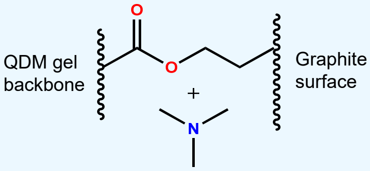
Gelatin	Graphite (+)	Oxidation	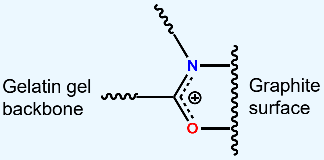
Gelatin	Graphite (-)	Reduction	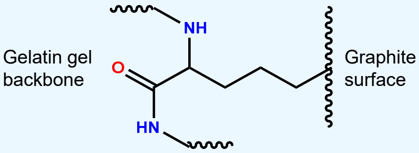

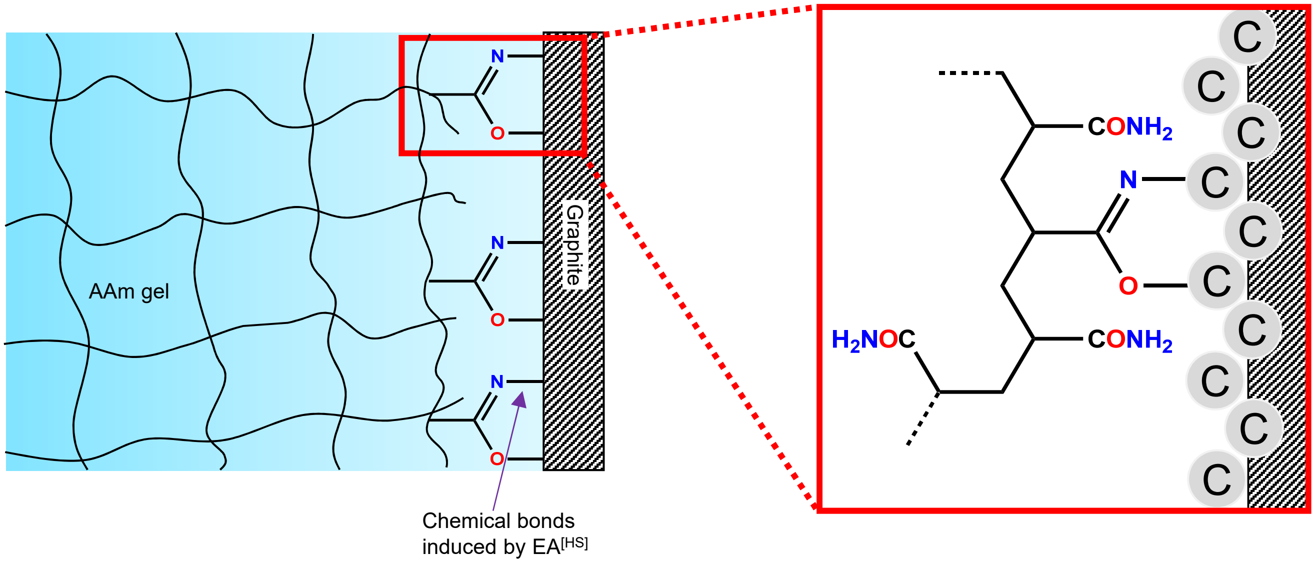

Supplement: Supplementary file 1 — oc3c01593_si_001.pdf [file oc3c01593_si_001.pdf]
