# Supplementary material for: Reversibly Sticking Metals and Graphite to Hydrogels and Tissues
Source: ACS Cent Sci. 2024 Mar 13;10(3):695–707. doi: 10.1021/acscentsci.3c01593 (PMC10979492; doi:10.1021/acscentsci.3c01593)
Supplement: Supplementary file 4 — oc3c01593_si_004.pdf [file oc3c01593_si_004.pdf]

oc-2023-01593b.R1

Name: Peer Review Information for "Reversibly Sticking Metals and Graphite to Hydrogels and Tissues"

First Round of Reviewer Comments

Reviewer: 1

Comments to the Author

The authors reported a simple method for adhering hard materials to soft, aqueous materials. This versatile adhesion phenomenon should be of interest to researchers in the fields of robotics, energy storage, and biomedical implants. I recommend the a minor revision of this manuscript.

The following issues still need to be discussed a little more for the applications in biomedical fields. For instance, the high voltage used in this experimental system is not safe for the biomedical implant; the surface contact with the electrode will be irreversibly damaged.

Reviewer: 2

Comments to the Author

The authors describe a very easy to implement, simple and amazingly universal method for inducing adhesion at the interfaces between solid conductors and ionic soft materials. The finding that the “hard-soft electroadhesion” can be induced so easily, and would provide strong, long-term bonding, which in most cases can be reversed as easily as it has been generated by reversing the voltage is surprising, very interesting and potentially very useful. It is also surprising that this phenomenon has not been investigated earlier, as it can be quite useful in many areas such as biomedical implants, soft robotics and soft materials structures. These findings could be useful for many investigators and may lead to lots of subsequent investigations, both on the origins and applications of this effect. The manuscript is certainly a good fit to CS due to its multidisciplinary implications. However, the paper needs revision to take into account the following.

1. The major way in which the paper can be enhanced is a more detailed and concrete data and interpretation of the chemical origins of the strong adhesion. The statement that the new effect

originates from electrochemical reactions that generate chemical bonds between the hard electrode and the gel network is likely correct. However, the exact reactions that occur at the interfaces remain unidentified. The authors present useful information on the correlation between the electrochemical potential and the adhesivity, FTIR data and suggest the type of groups and links in Table S2, but the reactions involved are speculative. Which of these reactions are precisely irreversible with the potential? It is recommended that the authors analyze in exact detail only two basic gel and electrode systems.

2. Some of the figure sections are relatively simple and conveying less important benchtop images. They could be moved to the SI. An example is Figure 5D, but others can be moved to the SI also.

3. “The fact that gelatin adheres to both electrodes also implies that this adhesion cannot be reversed by re-applying the field with reversed polarity.” This statement appears hypothetical and is not necessarily correct - depending on the mechanism and rates of adhesion and detachment this system may be temporarily reversible on one or both sides. Data on the reversibility in this case may provide interesting further information on the mechanisms involved.

4. Fig. 2(A). “Field strength” While the field at the electrode interface is correlated to the potential, the X-axis units are of potential, not field strength.

Author's Response to Peer Review Comments:

SRINIVASA R. RAGHAVAN
PROFESSOR and PATRICK & MARGUERITE SUNG CHAIR
Department of Chemical & Biomolecular Engineering
1227C BUILDING 090, COLLEGE PARK, MD 20742-2111

301-405-8164 PHONE
301-405-0523 FAX
sraghava@umd.edu
https://complexfluids.umd.edu

Senior Editor
ACS Central Science

February 2, 2024

Dear Editor,

Re: "Reversibly Sticking Metals and Graphite to Hydrogels and Tissues" (oc-202301593b)

Enclosed please find the revised version of the above paper. We are grateful to the reviewers for their comments. Our responses to the reviewers' comments and details of the changes to the manuscript are listed below. In addition, we have also attended to the items in the checklist that you had included.

Thank you for considering this manuscript. We hope you will find it acceptable for publication in *ACS Central Science*.

Yours sincerely,

Srinivasa R. Raghavan

Response to Reviewer 1

1. The authors reported a simple method for adhering hard materials to soft, aqueous materials. This versatile adhesion phenomenon should be of interest to researchers in the fields of robotics, energy storage, and biomedical implants. I recommend a minor revision of this manuscript.

   Thank you for the positive comments and the high ratings!

2. The following issues still need to be discussed a little more for the applications in biomedical fields. For instance, the high voltage used in this experimental system is not safe for the biomedical implant; the surface contact with the electrode will be irreversibly damaged.

   Our focus in this paper is not on biomedical applications. With that said, the conditions we employ for achieving electroadhesion (EA[HS]) can indeed be safe for future application to biological systems (e.g., sticking a metal to tissue). This is stated for the following reasons:

   First of all, the voltage used in EA[HS] is typically 5 to 10 V, which is much lower than the threshold to generate a significant electric shock (30 to 40 V). Secondly, when it comes to safety, the key variable is actually not the voltage, but the *current*. As long as the current is kept low, a living animal will not experience any adverse effects when a DC voltage such as the above is applied.

   As an example, we recently reported gel-to-tissue electroadhesion (Ref. 25) and we have tested this concept *in vivo* in live mice. We used voltages around 9 V to electroadhere a cationic gel to mouse intestines. The key was to keep the current values to ~ 1 to 2 mA, and if we did so, the mice were fine. We are writing up the results for publication.

Response to Reviewer 2

1. The authors describe a very easy to implement, simple and amazingly universal method for inducing adhesion at the interfaces between solid conductors and ionic soft materials. The finding that the "hard-soft electroadhesion" can be induced so easily, and would provide strong, long-term bonding, which in most cases can be reversed as easily as it has been generated by reversing the voltage is surprising, very interesting and potentially very useful. It is also surprising that this phenomenon has not been investigated earlier, as it can be quite useful in many areas such as biomedical implants, soft robotics and soft materials structures. These findings could be useful for many investigators and may lead to lots of subsequent investigations, both on the origins and applications of this effect. The manuscript is certainly a good fit to CS due to its multidisciplinary implications. However, the paper needs revision to take into account the following.

   Thank you for the kind words and the high ratings!

2. The major way in which the paper can be enhanced is a more detailed and concrete data and interpretation of the chemical origins of the strong adhesion. The statement that the new effect originates from electrochemical reactions that generate chemical bonds between the hard electrode and the gel network is likely correct. However, the exact reactions that occur at the interfaces remain unidentified. The authors present useful information on the correlation between the electrochemical potential and the adhesivity, FTIR data and suggest the type of groups and links in Table S2, but the reactions involved are speculative. Which of these reactions are precisely irreversible with the potential? It is recommended that the authors analyze in exact detail only two basic gel and electrode systems.

   We admit that the electrochemical reactions listed in Table S2 (i.e., those involved in inducing adhesion by EA[HS] at a solid-gel interface) are speculative.

   The system we have studied the most is the AAm-graphite system. We analyzed this system closely by FTIR (Figures 7 and S4). The spectrum for the AAm gel at the interface with the graphite anode (where adhesion by EA[HS] occurs) does show a new absorption peak at 1582 cm^{-1}. But we are hampered by the lack of information on this new FTIR peak from IR databases. However, this region of the IR spectrum seems to correspond to alkenes or aromatic rings. We have now mentioned this point in the revised manuscript.

   It was suggested to us that further insight could be gained by Raman spectroscopy. We have gone ahead and performed experiments using Raman confocal microscopy on the same AAm-

graphite system. The results are shown below. The AAm gel at the graphite anode (purple curve) does show a spectrum that is distinct from that for the AAm gel in the bulk. Indeed, this spectrum appears to be a superposition of the data for graphite alone (green curve) and AAm alone (orange curve).

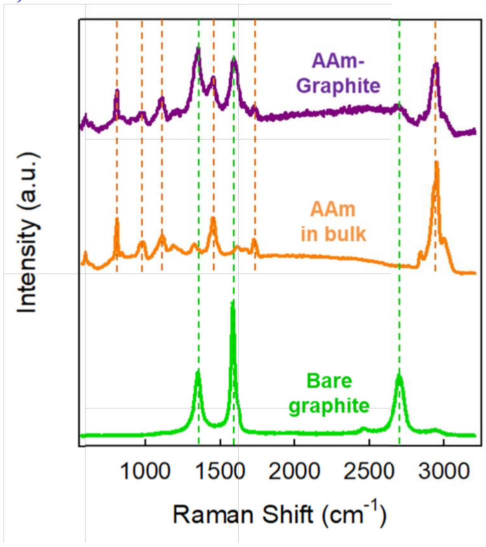

However, the above Raman spectrum does not show any visible new peaks that we can relate to specific bonds between the AAm gel and graphite at the interface. For this reason, we decided not to include the Raman data in the paper.

3. Some of the figure sections are relatively simple and conveying less important benchtop images. They could be moved to the SI. An example is Figure 5D, but others can be moved to the SI also.

   We thank the reviewer for the suggestion. We agree that Figure 5D may not be necessary and the same is also true for Figure 4D, both of which pertain to cases where there is no adhesion by EA[HS]. We have deleted both figures altogether from the paper and left the description of the results in words alone.

4. “The fact that gelatin adheres to both electrodes also implies that this adhesion cannot be reversed by re-applying the field with reversed polarity.” This statement appears hypothetical and is not necessarily correct - depending on the mechanism and rates of adhesion and detachment this system may be temporarily reversible on one or both sides. Data on the reversibility in this case may provide interesting further information on the mechanisms involved.

   This is an interesting point made by the reviewer. However, experimentally, we have not found the adhesion of gelatin to both graphite electrodes (anode and cathode) to be reversible. We have tried varying the times that the voltage is applied, but it has no discernible effect.

We have rewritten the above sentence in the text to make it clear that the lack of reversibility is indeed an experimental result, not just a hypothetical statement.

5. Fig. 2(A). “Field strength” While the field at the electrode interface is correlated to the potential, the X-axis units are of potential, not field strength.

   We have changed the heading of Fig. 2A to “Voltage” instead of “Field Strength”.
